# Semiconductor thermionics for next generation solar cells: photon enhanced or pure thermionic?

**DOI:** 10.1038/s41467-021-24891-2

**Published:** 2021-07-30

**Authors:** Ehsanur Rahman, Alireza Nojeh

**Affiliations:** 1 Department of Electrical and Computer Engineering, University of British Columbia, Vancouver, BC V6T 1Z4 Canada; 2 Quantum Matter Institute, University of British Columbia, Vancouver, BC V6T 1Z4 Canada

**Keywords:** Devices for energy harvesting, Solar energy, Electrical and electronic engineering

## Abstract

Semiconductors have been used in solar energy conversion for decades based on the photovoltaic effect. An important challenge of photovoltaics is the undesired heat generated within the device. An alternative approach is thermionics, which uses the thermal excitation of electrons from an emitter to a collector across a vacuum gap. If the emitter is a p-type semiconductor, the photogeneration-induced quasi-Fermi level splitting can reduce the effective barrier for electron emission—a mechanism used by a photon enhanced thermionic emission device. Here, we evaluate the prospects of this alternative solar conversion technology considering different semiconductor materials and thermionic device configurations. We also reveal that whether such a device operates in the photon enhanced or purely thermionic mode, depends on the complex interplay among materials properties, device physics and solar concentration level.

## Abstract (web summary)

A semiconductor thermionic device, which utilises thermally excited electrons, is considered as an alternative in solar conversion technology, yet its working mechanism is not clear. Here, the authors reveal that whether such a device operates in the photon enhanced or purely thermionic mode, greatly depends on the material properties and device physics.

## Introduction

Solar energy conversion is an important field of research due to solar radiation’s renewable nature and abundance as well as the rising environmental pollution concerns of conventional energy sources. At present, photovoltaics is the most widely used mechanism for generating solar electricity with demonstrated large scale implementation for both terrestrial and space applications. However, the efficiency of this technology is currently limited to around 20% for most practical systems. Although above-Shockley-Queisser performance has been demonstrated in multijunction photovoltaics and concentrated photovoltaics (CPV)^1^, these alternatives have not seen widespread utilization due to higher complexity and the associated costs. The primary challenges in photovoltaics can be traced to sub-bandgap photon loss and above-bandgap thermalization loss^2,3^. Even for a tandem photovoltaic cell, which can minimize the first loss by using multiple materials with different bandgaps, the second loss is inevitable, and the resulting heat is not only unutilized but also degrades the device performance^4^.

An interesting alternative to photovoltaics, which can turn this challenge into a benefit, is thermionic emission. Although thermionic energy converters (TECs) using metallic electrodes have been considered for solar conversion since the 1950s^5–8^, they eventually gave way to photovoltaics due to many other challenges^9–11^. A more recent development to solar thermionics is to utilize the very material used in photovoltaics (i.e., semiconductors). In particular, a photon enhanced thermionic emission (PETE) device^12–14^ is an insightful concept that uses a p-type semiconductor to combine the benefits of photovoltaics and thermionics: it takes advantage of the photogeneration-induced quasi-Fermi-level splitting to reduce the effective emission barrier for electrons while still delivering a high output voltage; it also utilizes the thermalization process to help electrons escape the material. Thus, the combined effects of light and heat on electron emission, studies of which go back to the works of Fowler and DuBridge^15–17^, has found a promising new application in solar energy conversion.

Taking advantage of this synergy between heat and light, PETE devices were estimated to outperform state-of-the-art solar cells, with an efficiency of around 40% for a single-stage device^13^ and higher than 50% with a second thermal cycle^13,18^. These early estimates were based on simplified models to show the potential of the concept. To study these devices in more detail, subsequent works have investigated some of the relevant physics such as thermal balance^19–21^, the space charge effect^20,22–24^, spatial variations of the charge carrier density^25–27^ and near-field radiative coupling^20,22^ to varying degrees (a detailed list of the different aspects of the physics treated in some of the previous key papers is provided in Supplementary Table 1). However, a comprehensive treatment of all the important materials and device physics has been missing. Owing to the complex interplay of the various pieces of physics involved, studying their individual contributions while simplifying other aspects does not enable a realistic analysis of these photothermal phenomena and devices based on them. As a result, fundamental questions still need to be addressed: whether or not a semiconductor thermionic device would operate in the PETE mode; and what the trends in device behavior and performance limits are if all the crucial physics are taken into account. Such a comprehensive study is the topic of the present work.

Our findings show that the device performance, while highly promising, is limited by the various tradeoffs in terms of material properties and device physics. Moreover, we reveal that the PETE mode is not guaranteed in a semiconductor thermionic solar cell under optimal operation, nor is it necessary for achieving a performance comparable to photovoltaics. This work sheds light on the issues and challenges in semiconductor thermionic solar conversion that need to be overcome when considering a complete device-level operation.

## Results

### Operation of a thermionic solar cell

**Fig. 1:**
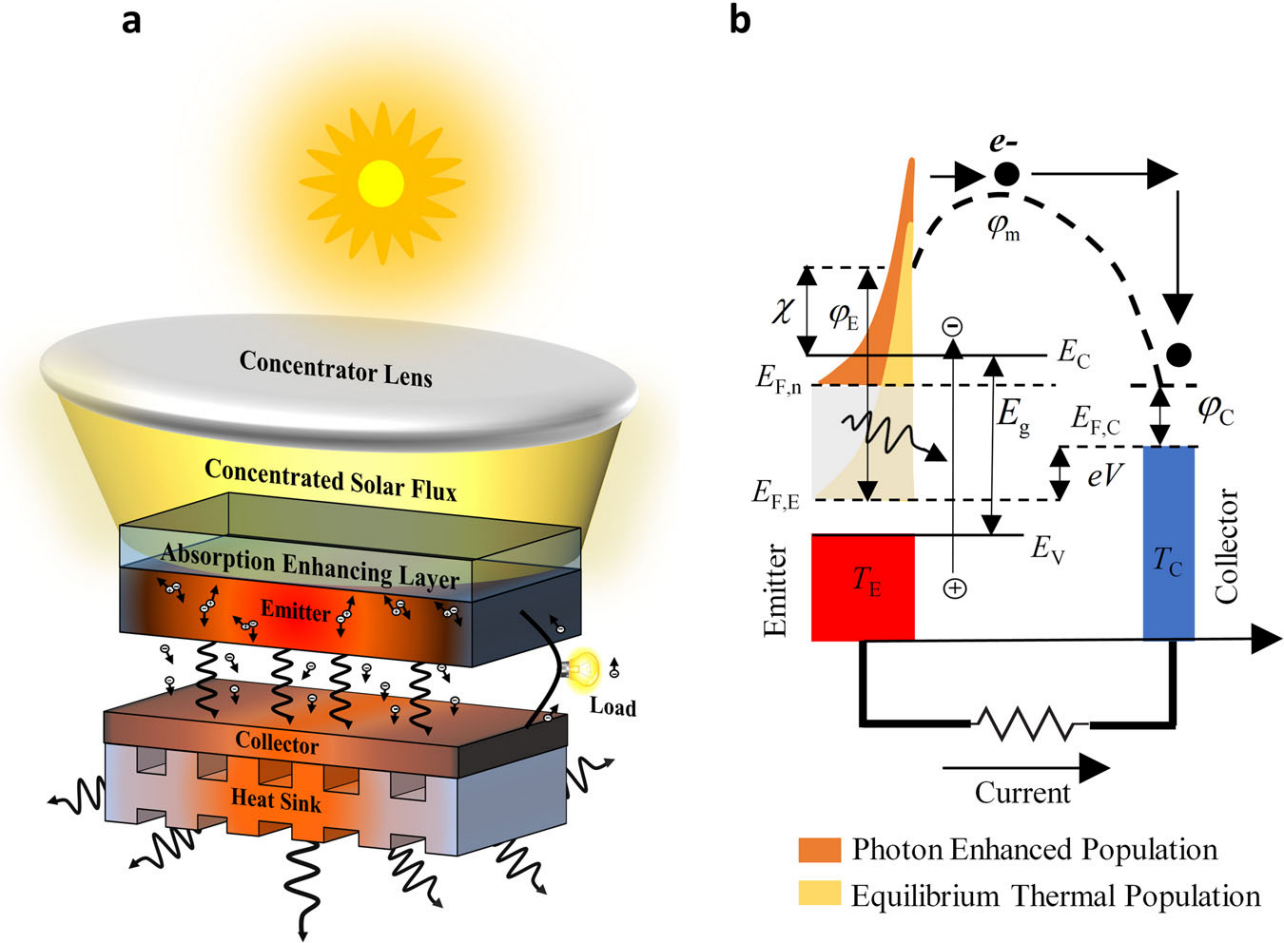
Operation of a semiconductor thermionic solar cell. **a** The schematic of a thermionic solar converter’s operation. **b** A simple band diagram of a semiconductor thermionic solar cell. *E*_F,E_ and *E*_F,C_ are the equilibrium Fermi levels in the emitter and collector, respectively, and *E*_F,n_ is the emitter quasi-Fermi level for electrons in the PETE mode. ${\varphi }_{{{{{{\rm{m}}}}}}}$ is the maximum motive in the interelectrode space and *e* is the electron charge. ${\varphi }_{{{{{{\rm{E}}}}}}}$ and ${\varphi }_{{{{{{\rm{C}}}}}}}$ are the work functions of the emitter and the collector, respectively. ${T}_{{{{{{\rm{E}}}}}}}$ and ${T}_{{{{{{\rm{C}}}}}}}$ are the temperatures of the emitter and the collector, respectively. ${E}_{{{{{{\rm{C}}}}}}}$, ${E}_{{{{{{\rm{V}}}}}}}$, and ${E}_{{{{{{\rm{g}}}}}}}$ are the conduction band minimum, the valance band maximum and the bandgap energy, respectively. *V* is the voltage difference between the two electrodes (and across the load).

Before delving into the details of materials and device physics and the associated challenges in a semiconductor thermionic solar cell, it is worth considering its basic operation with the help of a simple device schematic and its energy band diagram as shown in Fig. 1. A basic thermionic solar cell consists of two electrodes known as the emitter (or cathode) and collector (or anode) separated by a vacuum gap. In the emitter, the photogenerated electrons will undergo thermalization and various recombination processes, thereby broadening the thermal distribution of the electron population. Moreover, due to the photoexcitation of a large number of electrons, their thermal population may also be upshifted, which is the photon enhancement effect. The electrons in this excited thermal population that have sufficient energy to overcome the vacuum barrier will leave the material. These emitted electrons will be absorbed by the collector where they will thermalize again and do useful work with their remaining energy as they return to the emitter via an external circuit (the load receiving the generated power).

However, beyond this simple description, there are many intricate and interrelated pieces of materials and device physics that ultimately determine the operational mode and performance merits of a semiconductor thermionic solar cell. To investigate these effects, we have chosen silicon (Si) and gallium arsenide (GaAs), which have distinct properties. For example, Si, which is the most widely used material in photovoltaics, has an indirect bandgap and weaker optical absorption. On the other hand, GaAs is a direct-bandgap material with a sharp absorption onset near the Urbach edge^28,29^. Moreover, GaAs has a bandgap very close to the previously reported theoretical optimal value for PETE devices^13^. Also, the carrier diffusion lengths in these two materials are quite different, thanks to their vastly different recombination parameters^30^. There are also significant differences between their effective densities of states and dielectric properties. For both materials, we also study how the device configuration might affect the performance of a thermionic solar cell.

### Effect of cathode thickness

**Fig. 2:**
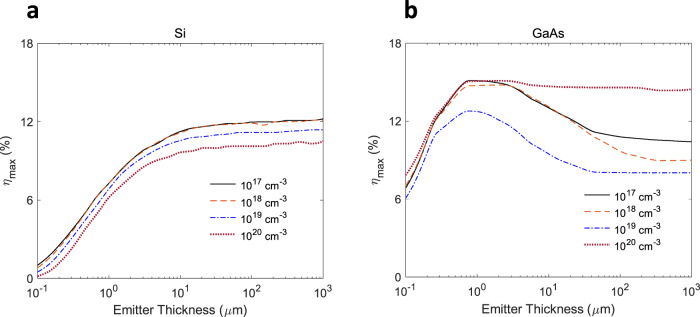
Variation of the solar conversion efficiency with emitter thickness. The data are shown at the maximum power point for **a** Si and **b** GaAs for different p-type doping levels in the emitter and a solar concentration ratio of 100.

First, we discuss how material properties affect the optimal emitter thickness and the mode of operation in a semiconductor thermionic solar cell. In general, the choice of the optimal thickness is dictated by the maximization of the absorption of the solar spectrum and minimization of the recombination of excess carriers during their transport to the emitting surface. These requirements are contradictory in terms of the material’s thickness. Based on our studies of different device configurations (which will be discussed later), we found that, under maximum conversion efficiency, Si maximizes the absorption of the solar spectrum. In other words, the nonradiative recombination-induced heating effect compensates for the carrier loss in Si at higher thicknesses (Fig. 2a) and results in a pure thermionic mode of operation. This finding is in contrast to the previous studies of Si cathodes where an optimal thickness for Si was predicted^26^, possibly due to the simplifications made in ref. ^26^ (see Supplementary Table 1). On the other hand, for GaAs, recombination loss becomes a limitation at larger thickness thereby resulting in a specific desired thickness that maximizes efficiency (Fig. 2b) by using the photon enhanced mode. Therefore, considering these aspects of recombination and optical absorption, the trends in device efficiency with the material’s thickness could vary from one semiconductor to another (Fig. 2).

The different behaviors seen in Fig. 2a, b with respect to doping level may be understood based on the fact that the electrode temperatures, optimal gap size, and optimal operating voltage are interdependent in a non-trivial way through energy balance and obtained self-consistently. At a fixed solar concentration, as the doping level in the GaAs cathode is increased from 10^17^ cm^−3^ to 10^20^ cm^−3^, the recombination probability increases, leading to higher cathode temperature. This in turn increases the radiative coupling, necessitating a wider interelectrode gap for the optimal operating point. The wider gap results in a higher space charge barrier, reducing the emission current. Therefore, with an increase in the doping level, initially the output power decreases. However, the increased doping level also leads to a higher optimal output voltage, and so the output power and efficiency eventually recover at the upper limit of the doping level. In the case of silicon, due to the lack of or weak photon enhancement effect at the optimal operating point (as will be seen later), the output voltage does not increase in the same manner at higher doping levels, and hence the trends in output power and efficiency are dominated by the trend in the current. For the rest of this study, we use a doping level of 10^18^ cm^−3^.

### Effect of interelectrode gap width and solar concentration

**Fig. 3:**
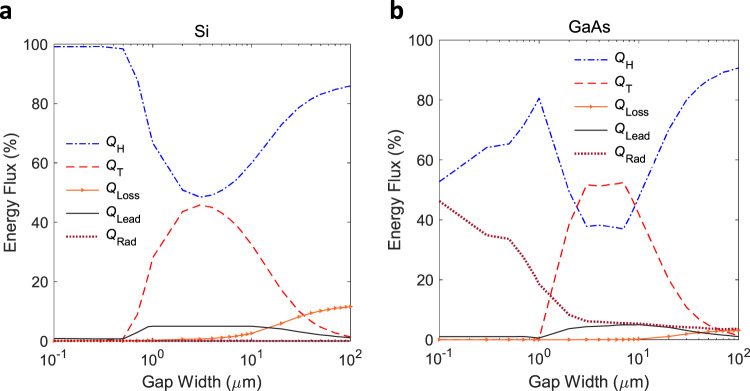
The interplay among different energy exchange channels in a semiconductor thermionic device. The data are shown as a function of the interelectrode distance (also referred to as gap width) for **a** Si and **b** GaAs at MPP under a solar concentration ratio of 100. The symbols (starting from the top) represent the interelectrode radiative and thermionic exchange, emitter thermal radiation loss to the ambient, net heat conduction through the lead and non-equilibrium radiative recombination loss, respectively.

We now discuss how the device configuration affects the semiconductor thermionic solar cell’s performance. The space charge effect is due to the Coulombic repulsion among the electrons transiting the device’s interelectrode space, which results in an additional energy barrier (Fig. 1b) for subsequent electron emission and transport^31,32^. Among the methods of mitigating this effect, using a micro-gap structure is in principle the simplest and results in a compact device with the fewest number of components. However, the micro-gap device performance is constrained by the near-field enhancement of interelectrode thermal radiative coupling. This near-field effect arises due to the coupling of evanescent waves between the electrodes when the gap width is comparable to the characteristic wavelength of thermal radiation (which is given by Wien’s displacement law and is of the order of a micrometer in practical temperature ranges)^33,34^. Besides, irrespectively of the gap size, there are additional energy loss mechanisms such as thermal radiation to the ambient and thermal conduction through the leads. The latter contains a tradeoff between the Joule heating effect and thermal conduction, leading to an optimal value for the lead resistance^31^. In a micro-gap device, the relative strength of these various energy exchange channels depends on the gap width and ultimately determines the electrode temperatures. This interplay among different energy exchange channels as a function of the gap width is shown in Fig. 3 at the maximum power point (MPP).

**Fig. 4:**
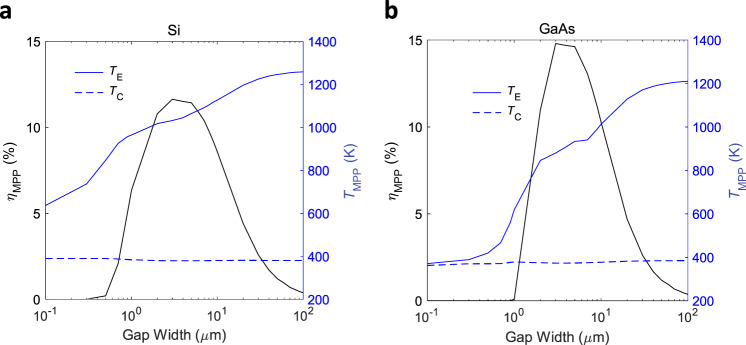
Trends in thermionic solar conversion efficiency and electrode temperatures as a function of the interelectrode gap width. The data are shown for **a** Si and **b** GaAs at MPP under a solar concentration ratio of 100. The symbols with subscript E and C represent the emitter and collector temperatures, respectively.

The corresponding efficiency and electrode temperature trends are shown in Fig. 4. The variations of these quantities’ gap width dependence with solar concentration are shown in the Supplementary Figs. 1–3. These strong dependencies of the energy exchange channels, electrode temperatures and conversion efficiency on gap width and solar concentration level demonstrate the importance of a complete account of the complex interdependencies of materials properties and device physics.

**Fig. 5:**
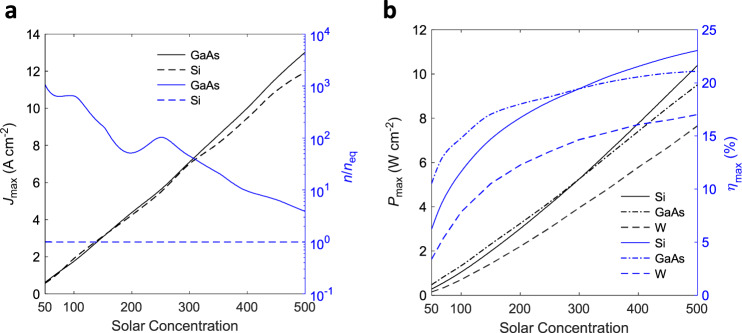
Performance metrics of different gap-optimized thermionic solar converters. **a** MPP current density and the corresponding photon enhancement factor (*n*/*n*_eq_) for different semiconductor materials as a function of solar concentration ratio. *n* and *n*_eq_ represent the conduction band electron density at the emitter surface under steady-state and equilibrium conditions, respectively. **b** MPP output power density and solar conversion efficiency for different semiconductor materials as a function of solar concentration ratio. The performance of a tungsten device is also shown for comparison between metal and semiconductor thermionics.

We now consider how the above tradeoffs translate to relevant performance metrics at the MPP for a wide range of solar concentrations (Fig. 5). Figure 5a shows the trend in the device current and the photon enhancement factor (*n/n*_eq_). The latter is a measure of the amount of optical upshift in the electron Fermi-level due to illumination, which can be written as

$${E}_{{{{{{\rm{F}}}}}},{{{{{\rm{n}}}}}}}-{E}_{{{{{{\rm{F}}}}}},{{{{{\rm{E}}}}}}}={k}_{{{{{{\rm{B}}}}}}}{T}_{{{{{{\rm{E}}}}}}}\,{{{{{\mathrm{ln}}}}}}(n/{n}_{{{{{{\rm{eq}}}}}}}),$$

where $n$ is the steady-state electron density at the emitter surface under photoexcitation and ${n}_{{{{{{\rm{eq}}}}}}}$ is the equilibrium electron density. ${k}_{{{{{{\rm{B}}}}}}}$ is the Boltzmann constant and ${T}_{{{{{{\rm{E}}}}}}}$ is the emitter temperature. The pure thermionic mode is defined as the case where the contribution from the excess electrons (under illumination) to the Fermi-level shift at the emitter surface is zero or negative (i.e., $n/{n}_{{{{{{\rm{eq}}}}}}}\le 1$).

Figure 5a also shows that, while GaAs takes advantage of photon enhancement and provides slightly higher current density at elevated concentration levels, Si prefers a pure thermionic mode. This challenges the prevalent assumption that PETE is the natural mode of operation of semiconductor thermionic devices. For example, in contrast to the present result, the simplifications made in ref. ^20^ led to the prediction of a high photon enhancement factor for Si cathodes. (The present model can reproduce the results of ref. ^20^ by incorporating similar simplifications, as shown by benchmarking in Supplementary Fig. 10.) We note that, even for GaAs, which tends to capitalize on the photon enhancement effect, this mode is not guaranteed: with the change of the collector material (which also changes the strength of interelectrode radiative coupling), even GaAs may prefer to operate in the pure thermionic mode (see Supplementary Fig. 4).

The non-monotonic, semi-plateau-like behavior of the photon enhancement effect in GaAs in Fig. 5a may be understood as follows. As the solar concentration is raised, the emitter temperature tends to rise, leading to a higher thermionic emission current and thus a stronger space charge effect. This is countered by a reduction in the optimal gap size, which in turn strengthens the near-field radiative coupling and thus opposes the rise in emitter temperature. Since the carrier densities depend on temperature, a signature of this behavior is also observed in the photon enhancement factor.

For comparison with conventional solar thermionics employing metal electrodes, in Fig. 5b we also show the conversion performance using tungsten electrodes and a selective tungsten pyramid solar absorber^35^ (this comparison is motivated by the long history of solar thermionics using metallic emitters, which dates back to the 1950s; it is intriguing to know where the relatively new concept of semiconductor thermionics stands in comparison to its metal counterpart). Interestingly, Fig. 5b shows that even without leveraging the photon enhancement effect, Si can outperform GaAs for higher concentration levels in terms of overall conversion performance. These findings signify that, although the PETE mode is in principle desirable, achieving this mode is not trivial in all semiconductor devices, nor is it necessary at all concentration levels. We also note that the efficiency predictions for GaAs at higher concentration levels may not be achievable in practice as that material begins to decompose well below its melting point.

**Fig. 6:**
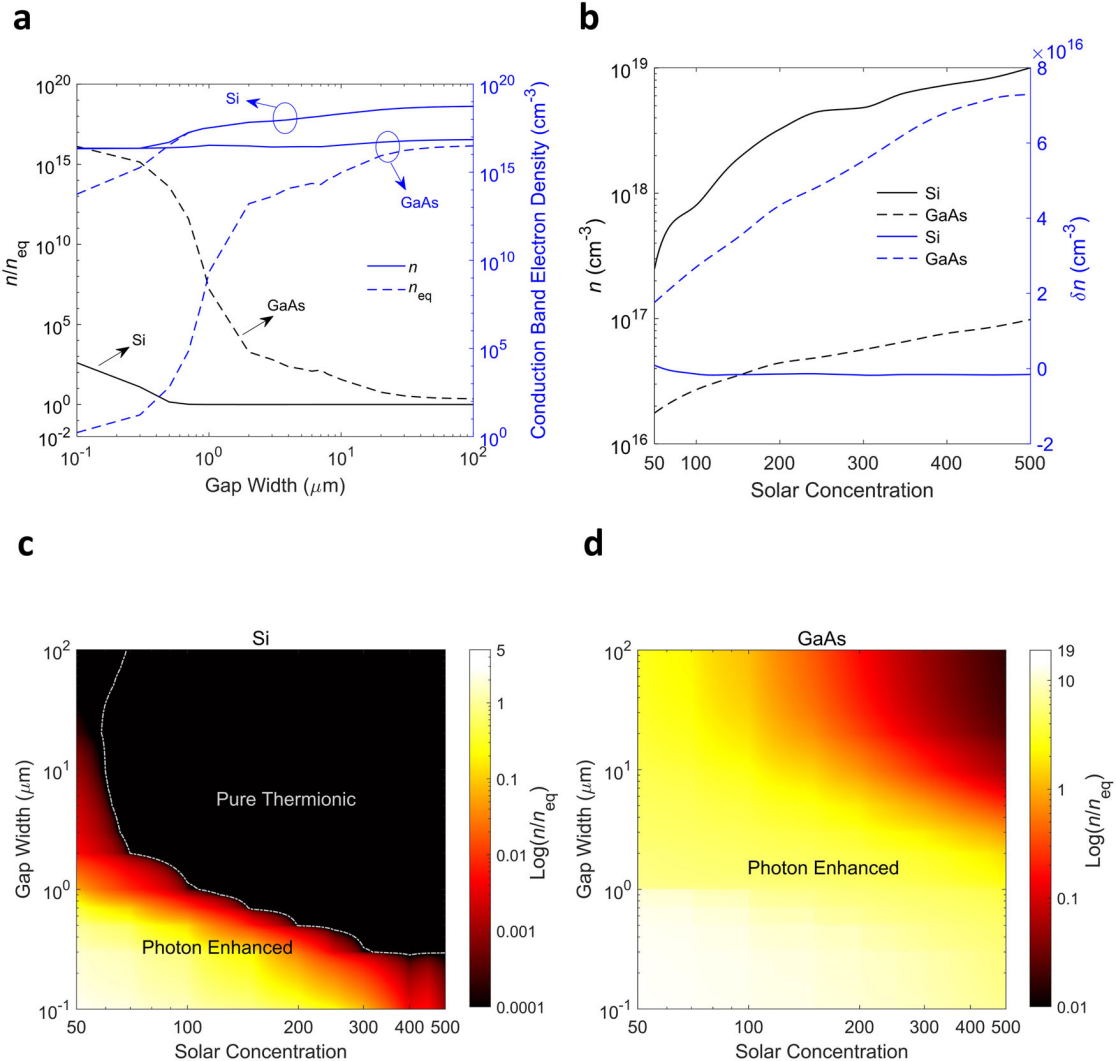
Trends of conduction band electron density and contributions from photogeneration at the emitter surface with gap width and solar concentration ratio. **a** Conduction band electron density (equilibrium (*n*_eq_) and steady-state (*n*)) and the associated photon enhancement factor (*n*/*n*_eq_) as a function of interelectrode gap width. The data are shown at MPP and for a solar concentration ratio of 100. **b** Steady-state and excess carrier density ($\delta n$) in the conduction band for different semiconductor materials as a function of solar concentration ratio at MPP. Photon enhancement factor for **c** Si and **d** GaAs as a function of interelectrode gap width and solar concentration ratio. The data are shown at MPP and the dash-dotted line indicates the boundary between the PETE and pure thermionic regimes.

To gain further insight into these two materials’ different behaviors concerning the photon enhancement effect, in Fig. 6a, b we show the trends in steady-state conduction band electron density at the emitting surface together with the contribution from photogeneration (under MPP), as a function of both the gap width and the solar concentration level. It is worth noting that the dependence of the mode of operation on gap width is not trivial. As the gap width is increased, the cathode temperature increases (see Fig. 4a, b) due to the net result of the interplay among the energy exchange channels. This will increase the thermal generation of electron-hole pairs and the associated recombination probability, which ultimately determine the steady-state carrier density in the cathode at the emitting surface, which in turn informs the mode of operation.

Interestingly, while the total carrier concertation shows an upward trend for both materials (for the trend with gap width, this is due to a stronger thermal generation at elevated temperatures with the increase of gap width; for the trend with solar concentration, this is due to both higher photon flux and increased thermal generation with increasing concentration level), the excess carrier contribution shows completely different trends for the two materials considered (Fig. 6b). Regarding the trend versus gap width, for Si, the excess carrier contribution is negligible over most of the gap range (a signature of the pure thermionic mode of operation), whereas for GaAs, this contribution, while still being positive, gradually decreases with increasing gap width. We attribute these differences to the varying degrees of thermal generation enhancement and the associated increase in recombination probability as well as the different thickness optimization criteria for these two materials. The trends with increasing solar concentration level can be explained by similar reasoning. The combined effect of the gap width and solar concentration level on the photon enhancement factor is shown in Fig. 6c, d).

### Micro-gap and macro-gap device performance comparison

**Fig. 7:**
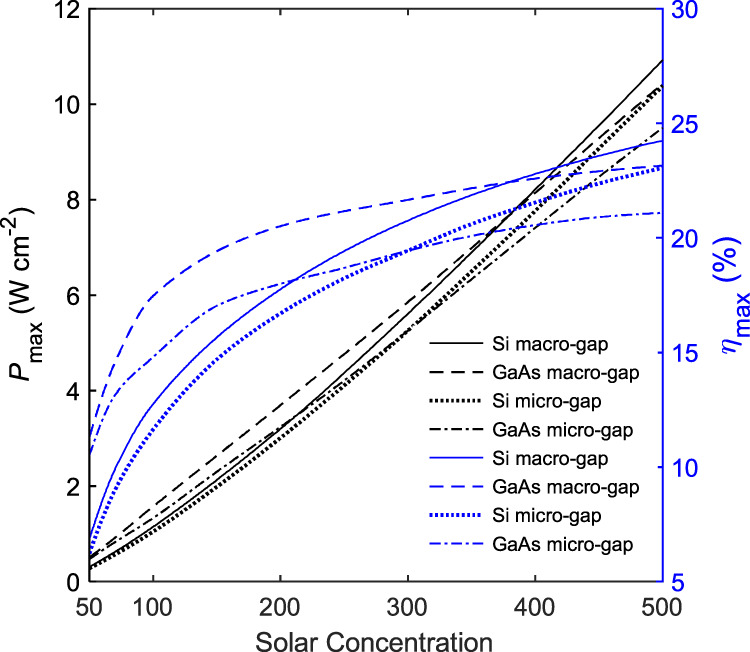
Performance comparison between different thermionic device configurations. At each solar concentration ratio, the data are shown at MPP, and the optimal interelectrode gap width was chosen for the micro-gap devices. For macro-gap devices, the interelectrode gap width is of the order of a millimeter.

Finally, we note that there exist alternative TEC structures that also mitigate the space charge effect: the vapor TEC involves the inclusion of positive ions (such as Cs^+^)^31^, which will neutralize the negative charge of the transiting electrons, while the triode TEC uses a gate electrode between emitter and collector to prevent the buildup of a negative charge cloud^36,37^. These structures, although involving their own specific loss mechanisms, do not need small interelectrode gaps and hence do not incur near-field radiative loss. Therefore, it is in principle possible for them to enable higher performance compared to micro-gap devices. Within our model, we can gain insight into the upper limit of performance for these devices by neglecting the loss mechanisms associated with space charge and its mitigation strategy. For the materials studied in this work, the performance improvement resulting from such macro-gap architectures was found to be marginal (Fig. 7) (this indicates that, in fact, for these two materials, at the optimal gap size, the near-field radiative coupling in the micro-gap structure was already minimal). It is worth noting that these alternatives also involve other challenges. For example, a vapor TEC’s lifetime is limited by the availability of the ions, and ion deposition might result in unwanted secondary electron emission sources; in a triode TEC, gate leakage can reduce conversion efficiency^31^ and the device also requires additional circuitry to set up the gate voltage and magnetic fields to prevent electrons from striking the gate^37^. Note that the results shown in Fig. 7 are based on a best-case estimate for macro-gap devices, and even then these devices do not present a significant advantage over the micro-gap device for the materials considered.

Figure 7 also shows that, under optimal operation and for different device structures, the realistic thermionic solar cell efficiencies for the two semiconductors studied (which are also widely used in photovoltaics) are comparable to those of commercial single-junction photovoltaic cells at the higher concentration levels. To further improve the device performance, a second thermal cycle can be implemented, which would utilize the waste heat released from the collector. This second stage can be a thermoelectric, thermophotovoltaic or any other type of heat engine. With such hybrid operation, the conversion efficiency can be comparable to the multijunction and CPV performance and can exceed the Shockley-Queisser limit^38^. This hybrid generation capability is an additional advantage of thermionic and PETE devices due to their high temperature operation.

## Discussion

Based on the above results and analyses, we conclude that while thermionic conversion using semiconducting emitters is a promising path and can address the thermal limitation of photovoltaics, its overall performance is still directly limited by materials and physics-related challenges. Moreover, beyond those fundamental issues, additional difficulties need to be overcome for thermionics to compete with photovoltaics. Here we point out these issues and possible solutions to both fundamental and practical challenges, in order to provide a broader perspective as well as to motivate further research into semiconductor thermionics.

First, the high temperature stability of various electrodes, surface coatings and electrical contacts needs to be ensured (in addition, contact geometry needs to be optimized for maximum access to sunlight; the contact design technologies from existing CPV systems may be of help in this regard). Also, the emission and collection probability of the thermally excited electrons need to be increased as much as possible, and surface treatment may provide a solution. For example, the Richardson constant of nitrogen-incorporated diamond films was improved by four orders of magnitude via hydrogen plasma treatment^39^. Additionally, surface recombination needs to be minimized (unless the device is operating in the pure thermionic mode). This may be achieved by adding an energy barrier near the emitting surface such as by creating a heterostructure^40^. In addition, trap-assisted recombination can be detrimental to the photon enhancement effect. This carrier loss mechanism strongly depends on the growth process of the material and material handling during device fabrication steps^41^, and needs to be minimized.

Manipulating the material dimensionality via micro- and nano-fabrication techniques may result in improved material properties such as increased optical absorption, higher electron emission probability, reduced thermal conductivity, etc. For example, carbon nanotube arrays with long-range alignment (CNT forests), grown using chemical vapor deposition, exhibit near-perfect optical absorption over a wide spectral range^42^ and efficient heating and multiphoton photothermal emission^43,44^. Semiconducting CNTs that may exploit the PETE mechanism can be created by controlling the nanotube chirality. However, the CNT work function is typically above 4.5 eV and needs to be reduced through coatings with high temperature stability^45,46^. Vertically aligned III–V nanowire arrays have also shown low reflectance over the visible spectrum, which can be tuned by adjusting the nanowire diameter^47^ or the growth time^48^. The ultimate challenge is to combine all the desired properties into a single material or heterostructure.

Overall, the concept of semiconductor thermionics is still in early stages and much remains to be investigated on the experimental front. However, such experimental and design efforts also require a comprehensive understanding of this conversion mechanism and its underlying physics. Therefore, in this work, we revealed the effects of these materials and device physics, providing realistic performance estimates for devices based on widely used semiconductors.

## Methods

### Overview of the modeling approach

For the analysis of the semiconductor thermionic solar cell carried out in this work, we took into account the spatial dependence of optical absorption and transport of the photogenerated carriers within the emitter. Charge carrier transport inside the cathode, in general, involves both drift and diffusion. However, in cases where the electric field inside the cathode is negligible, the drift component can be neglected and the carrier balance is dictated by the diffusion mechanism. A discussion on these mechanisms as relevant to the present study can be found in Supplementary Note 1. Electron transport in the interelectrode space and the associated space charge effect were incorporated using a phase space formalism. The interelectrode radiative exchange was calculated using fluctuational electrodynamics and the electrode temperatures were calculated using complete energy balance. An overview of the implementation of these physics is discussed below, and the detalied formulation is presented in the Supplementary Information.

### Cathode

The absorption profile of the solar spectrum and the associated spatial variations of the photogenerated electron-hole pairs in the semiconductor emitter were analyzed using the particle continuity equation that governs the generation, recombination and transport of the charge carriers. The various radiative and nonradiative recombination mechanisms were implemented using theories that are valid under both low and high injection levels. The associated recombination coefficients and lifetimes were taken from the literature. The detailed implementation of the cathode model is discussed in Supplementary Note 1. For Si (where the solar conversion performance monotonically improves with the thickness), the material’s thickness was taken to be 20 µm (this thickness was chosen so that at least 80% of the solar spectrum is absorbed). Also, the resulting efficiency almost saturates at this thickness, justifying saving on additional computational expenses associated with larger thicknesses. For GaAs, the thickness was taken at its optimal value (Fig. 2b) for the doping level considered. For emitter electron affinity, we considered a value of 1 eV (obtainable through appropriate surface coating) and the theoretical value of 120 Acm^−2^ K^−2^ was used for Richardson’s constant for thermionic current calculations.

For incident solar radiation, we considered the AM 1.5 direct plus circumsolar spectrum concentrated by different concentration ratios used in this study. The upper level of the solar concentration ratio used (500x) is based on practically achievable values, as shown in both commercial and laboratory-based CPV systems^1^. We have chosen p-type doping in the emitter to maximize the photon enhancement effect in case this mode occurs during the solar cell’s operation. For the collector, we considered a heavily n-type doped semiconductor made of the same material as the emitter. For the analyses shown in Figs. 3–7, we considered a p-type doping level of 10^18^ cm^−3^ in the cathode. The anode work function was taken to be 1 eV. For the study of metal thermionics, we considered tungsten electrodes due to the material’s high melting point. As the intrinsic work function of tungsten is too high to obtain significant thermionic emission at practically achievable temperatures, we assumed a barium-activated tungsten cathode^49^ and a Cs-activated tungsten anode^50^. The heat transfer coefficient of the anode to the heat sink was taken as 0.1 Wcm^−2^, which represents the upper limit of cooling by free convection.

### Space charge and near-field radiative coupling

The space charge effect was treated by solving the coupled Poisson-Vlasov equations. The related phase space analysis of the thermionically emitted electrons in the space charge mode is discussed in the Supplementary Note 2. The interelectrode thermal radiative coupling was calculated using fluctuational electrodynamics. This ab initio method accounts for the near-field coupling of the evanescent waves as well as the far-field propagating waves, and the interference of the thermally generated electromagnetic waves in the device’s interelectrode space. The detailed implementation of this model is discussed in the Supplementary Note 3.

### Semiconductor material properties

The semiconductor material’s properties such as spectral absorptivity, reflectivity and electron and hole mobilities were taken from various experimentally validated models considering their temperature and doping dependencies. The dielectric permittivities (which are needed for optical absorption and thermal radiation calculations) of the materials were taken from various empirical models considering their temperature and doping dependencies. These dielectric models are discussed in the Supplementary Note 4. The density of states and conductivity effective mass for the materials were taken from the literature. The temperature dependence of the effective density of states was considered. The temperature-induced bandgap narrowing effect was considered using the empirical Varshni relation. The equilibrium Fermi level was calculated using the charge neutrality criterion with experimentally reported energy levels for the shallow dopants.The relevant details of the theories that describe the abovementioned material properties are discussed in Supplementary Note 5, with references given therein.

### Self-consistent calculation of particle and thermal balance

The electrode temperatures were calculated by solving the coupled energy and particle balance criteria in a self-consistent iterative process. The detailed implementation of this self-consistent algorithm is discussed in Supplementary Note 6.

## Supplementary information

Supplementary Information

## Data Availability

The numerical data that support the findings of this study are available in “figshare” repository (10.6084/m9.figshare.14818590.v1)^51^.

## Code availability

The codes that support the findings of this study are available from the corresponding author upon reasonable request.
