# Supplementary material for: Semiconductor thermionics for next generation solar cells: photon enhanced or pure thermionic?
Source: Nat Commun. 2021 Jul 30;12:4622. doi: 10.1038/s41467-021-24891-2 (PMC8324797; doi:10.1038/s41467-021-24891-2)
Supplement: Supplementary file 1 — Supplementary Information [file 41467_2021_24891_MOESM1_ESM.pdf]

SUPPLEMENTARY INFORMATION

Semiconductor Thermionics for Next Generation Solar Cells: Photon Enhanced or Pure Thermionic?

Ehsanur Rahman[1,2]* and Alireza Nojeh[1,2]

[1] Department of Electrical and Computer Engineering, University of British Columbia, Vancouver, BC, V6T 1Z4, Canada

[2] Quantum Matter Institute, University of British Columbia, Vancouver, BC, V6T 1Z4, Canada

*Email: ehsanece@ece.ubc.ca

Supplementary Tables

Supplementary Table 1. Materials and device physics implemented in the present work compared to past literature

Modelling Aspects	[1] Schwede *et al.* [2010]	[2] Segev *et al.* [2012]	[3] Varpula *et al.* [2012]	[4] Segev *et al.* [2013]	[5]Su *et al.* [2013]	[6]Sahasrabuddhe *et al.* [2013]	[7] Su *et al.* [2014]	[8] Varpula *et al.* [2015]	[9] Segev *et al.* [2015]	[10] Wang *et al.* [2019]	[11] Liu et al. [2019]	This Work
Emitter particle balance	Spatial Variation Neglected	Spatial Variation Neglected	1D Analytical Diffusion Model	1D Numerical Drift-Diffusion Model	Spatial Variation Neglected	1D Analytical Diffusion Model<br><br>Note: This work is an emitter model, not a complete device model.	Spatial Variation Neglected	1D Numerical Diffusion Model	Spatial Variation Neglected	Spatial Variation Neglected	Spatial Variation Neglected	1D Numerical Drift-Diffusion Model
Thermal balance in both electrodes	X	Emitter only	X	Emitter only	√	Not Applicable (NA)	X	X	Emitter only	Emitter only	X	√
Electron recycling	X	√	√	√	X	NA	X	√	√	√	X	√
Space charge effect	X	X	X	X	X	NA	√	X	√	√	√	√
Near-Field coupling	X	X	X	X	X	NA	X	X	X	√	√	√
Recombination mechanisms	Radiative only	Radiative only	Bulk & Surface (low-level injection)	Bulk & Surface	Radiative only	NA	Radiative only	Bulk & Surface (low-level injection)	Radiative only	Radiative only	Radiative only	Radiative and non-radiative bulk & surface (low and high-level injection)
Temperature and doping dependence	Density of states (DOS)	X	To some extent	To some extent	DOS	NA	DOS	To some extent	X	X	X	√
Realistic optical properties of the material for radiative calculation	X<br><br>Note: An abstract notion of IR absorber was used.	X<br><br>Note: An abstract notion of IR absorber was used.	To some extent	X<br><br>Note: An abstract notion of IR absorber was used.	X	NA	X	To some extent	X	To some extent	To some extent	√
Thermal loss in lead and the related optimization	X	X	X	X	X	NA	X	X	X	X	X	√

Supplementary Table 2. Material and device parameters used in the study

Parameters	Si	GaAs
Electron Auger recombination coefficient ($cm^6 s^{-1}$)	** 1.1×10^{-30} [12]	1.6×10^{-29} [8]
Hole Auger recombination coefficient ($cm^6 s^{-1}$)	** 3×10^{-31} [12]	4.6×10^{-31} [8]
Shockley-Reed-Hall lifetimes (s)	** [13]	** [14]
Front surface recombination velocity ($cm\ s^{-1}$)	100 [8]	100 [8]
Back surface recombination velocity ($cm\ s^{-1}$)	0 [8]	0 [8]
Temperature dependence of bandgap narrowing effect (eVK^{-1})	4.73×10^{-4} [12]	5.405×10^{-4} [12]
Bandgap at 0K (eV)	1.17 [12]	1.519 [12]
Electron density of state effective mass	1.18 [12]	0.0708 [15]
Hole density of state effective mass	0.81 [12]	0.53 [12]
Carrier mobility	** [16]	** [17]
Dopant ionization energy (eV)	0.045 [12]	0.02 [12]
Optical absorption coefficient	** [18,19]	** [8,20]
Heat transfer coefficient of the heat sink ($W\ cm^{-2} K^{-1}$)	0.1 [21]	
Richardson constant ($A\ cm^{-2} K^{-2}$)	120	
Emitter electron affinity (eV)	1	
Collector work function (eV)	1	
Micro-gap device gap size range (μm)	0.1−100	
Macro-gap device gap size (mm)	1	
Solar concentration ratio	50× ~ 500×	

** Dependencies are modelled from the cited references.

Supplementary Figures

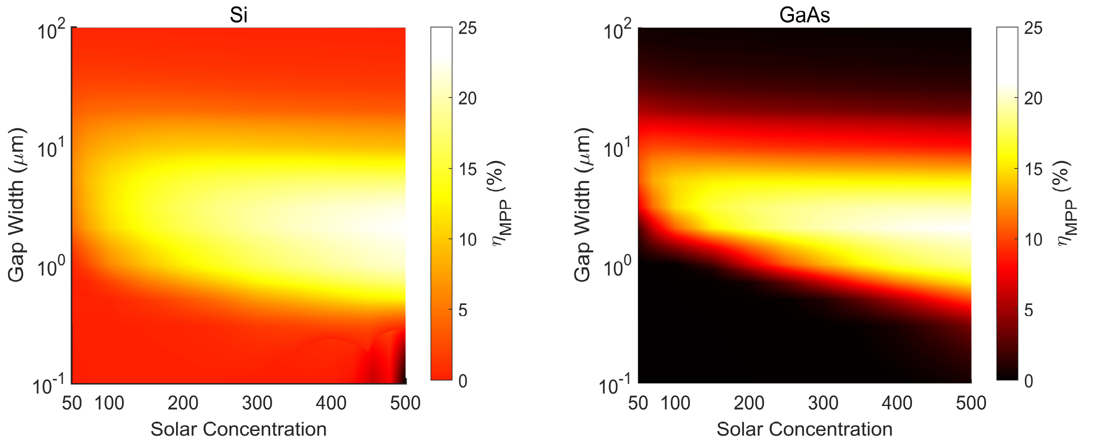

(a) (b)

Supplementary Fig. 1. MPP conversion efficiency for (a) Si and (b) GaAs as a function of the interelectrode gap width and solar concentration ratio.

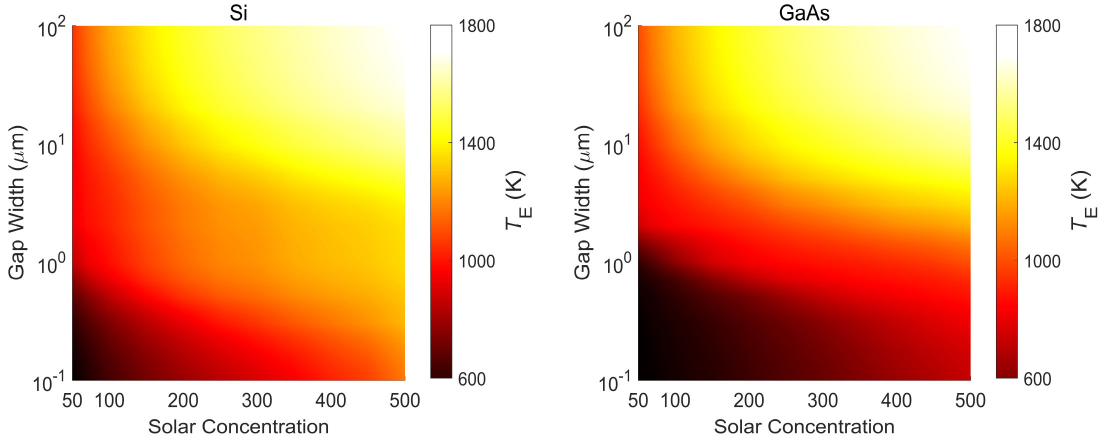

(a) (b)

Supplementary Fig. 2. Emitter temperature for (a) Si and (b) GaAs as a function of the interelectrode gap width and solar concentration ratio. The data are shown at MPP.

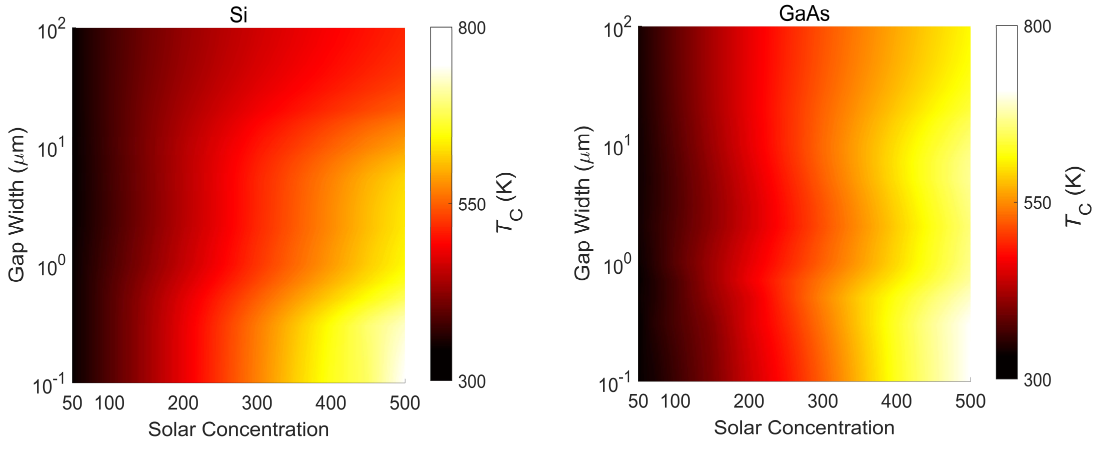

(a) **(b)**

Supplementary Fig. 3. Collector temperature for (a) Si and (b) GaAs as a function of the interelectrode gap width and solar concentration ratio. The data are shown at MPP.

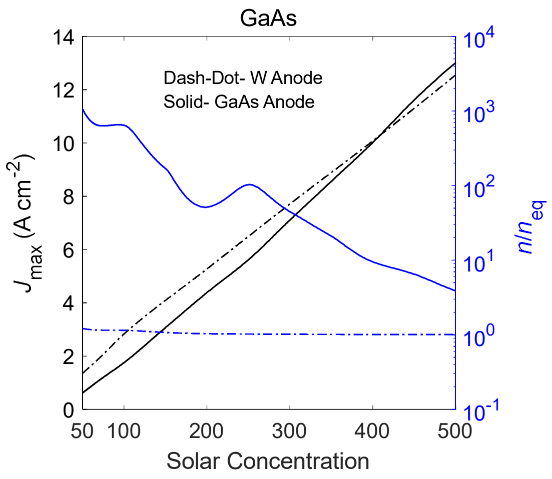

Supplementary Fig. 4. The variation of the photon enhancement factor and current density with solar concentration ratio under the optimal operating condition for a micro-gap thermionic solar cell. The data are shown for the GaAs emitter and n-type GaAs (solid line) collector or W (dash-dotted line) collector. The device parameters are similar to that of the GaAs emitter study shown in Fig. 5(a) of the main manuscript.

Supplementary Computational Methodologies

Supplementary Note 1. Semiconductor emitter model for calculating the spatial distribution of solar absorption and photogenerated electron-hole pairs

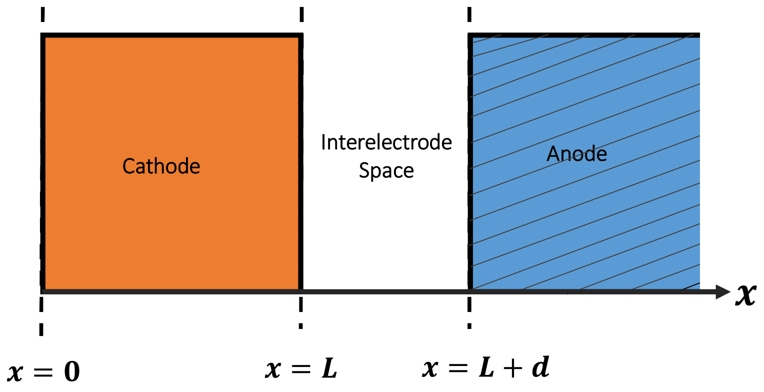

Supplementary Fig. 5. A simple illustration of the various dimensions of the device components. L is the thickness of the emitter (cathode) and d is the interelectrode gap width.

The spatial distribution of the electrons, $n(x)$, and holes, $p(x)$, inside the emitter (under photogeneration) can be obtained by solving the drift-diffusion equations for the electrons and holes, respectively, which can be written as

$$\nabla.\mathbf{J_n} = -(G-R), \qquad (1)$$
$$\nabla.\mathbf{J_p} = (G-R), \qquad (2)$$
$$\mathbf{J_n} = \mu_n n \mathbf{E_x} + D_n \nabla n, \qquad (3)$$
$$\text{and } \mathbf{J_p} = \mu_p p \mathbf{E_x} - D_p \nabla p. \qquad (4)$$

In the above equations, $\mathbf{J}_n$ and $\mathbf{J}_p$ are the particle fluxes for electrons and holes, respectively; G and R are the optical generation and recombination rates (the modelling of which will be discussed later in this section), respectively; $\mathbf{E_x}$ is the electric field and D_n, D_p are the diffusion coefficients for electrons and holes, respectively. To solve the above-mentioned drift-diffusion equations, the boundary conditions for the electron and hole particle fluxes at both surfaces of the emitter are needed. At the front surface (*i.e.* at $x = 0$), the boundary conditions are

$$J_n(0) = S_{n,F}\delta n(0) \qquad (5)$$
$$\text{and } J_p(0) = -S_{p,F}\delta p(0) \qquad (6)$$

, where $S_{n,F}$ and $S_{p,F}$ are the front surface recombination velocities for electrons and holes, respectively.

On the other hand, at the back surface (*i.e.* at $x=L$), the boundary conditions can be written as

$$J_n(L) = -S_{n,B}\delta n(L) - J_{net} / e \qquad (7)$$
$$\text{and } J_p(L) = S_{p,B}\delta p(L) \qquad (8)$$

, where $S_{n,B}$ and $S_{p,B}$ are the back surface recombination velocities for electrons and holes, respectively and J_{net} is the net thermionic current density between the emitter and collector. The electric field inside the emitter can be obtained by solving Poisson's equation, which can be written as

$$\frac{d^2\psi_{Emitter}}{dx^2} = -\frac{e}{\varepsilon_{Emitter}}(p - n - N_A^- + N_D^+) \qquad (9)$$

, where N_A^- and N_D^+ are the ionized acceptor and donor concentrations, respectively and $\varepsilon_{Emitter}$ is the permittivity of the emitter.

In solving the above-mentioned Poisson's equation in the emitter, the Dirichlet boundary condition is used at the front surface and the Neumann boundary condition is used at the back surface, which can be written as

$$-\varepsilon_{Emitter}\frac{d\psi_{Emitter}}{dx}\Big|_{x=L} = -\varepsilon_0\frac{d\psi_{Interelectrode}}{dx}\Big|_{x=L} \qquad (10)$$

, where ε_0 is the permittivity of the free space.

Note that the above-mentioned coupled drift-diffusion and Poisson's equations need to be solved iteratively until convergence is achieved, which is highly computationally expensive and time-consuming. The computational algorithm for this iterative analysis is provided in Supplementary Note 6.

However, the particle balance problem can be simplified assuming negligible electric field inside the emitter, which we will refer to as the zero-field approximation. Under this approximation, the transport of the electrons is uncoupled from that of the holes and the carrier

transport is dominated by the diffusion mechanism. This is a valid approximation for a highly doped p-type emitter where the high concentration of majority carriers (holes) can neutralize any charge imbalance that could produce any significant electric field inside the emitter. To verify the suitability of the latter approach for the device operating conditions considered in this work, we have initially investigated the device operation where the particle balance problem has been solved using both the drift-diffusion and negligible field approaches. A comparison between the findings from these two approaches is shown in Supplementary Table 3 for the micro-gap device under optimal operation. For this comparative study, we have used the same doping concentration and emitter thickness considered in Figs. 3-6 of the main manuscript.

Supplementary Table 3. Comparison between the drift-diffusion and zero-field approximations

Particle balance modeling approach	Current Density (Acm^{-2})				Power Density (Wcm^{-2})				Efficiency (%)				Mode of Operation			
	Si		GaAs		Si		GaAs		Si		GaAs		Si		GaAs	
	100x	500x	100x	500x	100x	500x	100x	500x	100x	500x	100x	500x	100x	500x	100x	500x
Drift-diffusion	2.01	12.55	1.73	13.50	0.95	9.06	1.31	8.69	10.52	20.14	14.58	19.30	PT	PT	PE	PE
Neglecting the field within cathode	1.90	11.94	1.76	13.02	1.049	10.39	1.33	9.50	11.64	23.04	14.79	21.09	PT	PT	PE	PE

Note: In the above table, PT stands for the pure thermionic mode and PE stands for the photon enhanced thermionic mode.

From these comparisons, we see that, quantitatively, the zero-field approximation slightly overestimates the device performance compared to the drift-diffusion approach, which is expected due to the more rigorous analysis of the emitter electric field and the associated Ohmic loss. However, these quantitative changes do not make any differences in the fundamental conclusions. For example, using both approaches, we have found that the Si device operates under the pure thermionic mode whereas the GaAs device operates under the photon enhanced thermionic mode. Therefore, to save on excessive computational expenses and keep the computational time manageable during the parametric variation studies of the gap width and solar concentration level, we have assumed zero field inside the emitter for the findings shown in the main manuscript. (Note that the more rigorous approach shown above can be used as appropriate for cases where the zero-field approximation is not a good assumption). The details of the zero-field approach will be discussed below.

Assuming zero field inside the emitter, the spatial distribution of the electrons inside the emitter, $n(x)$, can be obtained from the particle continuity equation as [15]

$$D_{\mathrm{n}} \frac{d^2 n}{dx^2} = R(x) - G(x) \ . \qquad (11)$$

The recombination rate consists of radiative and various non-radiative mechanisms (Shockley-Reed-Hall, Auger and surface). On the other hand, the generation rate depends on the photon absorption profile, which can be written as [8]

$$G(x) = \int_0^{\lambda_g} \phi \ \alpha \ (1-\rho_{\mathrm{F}}) \ [\exp(-\alpha x) + \rho_{\mathrm{B}} \exp(-\alpha(2L - x))] \ d\lambda \ . \qquad (12)$$

In the above equation, λ is the photon wavelength, α is the absorption coefficient, ϕ is the incident flux of photons, ρ_{F} is the reflection coefficient of the irradiated front surface (*i.e.* the surface where the light enters the emitter), ρ_{B} is the reflection coefficient of the electron-emitting back surface, and L is the thickness of the emitter. In general, α, ϕ, ρ_{F}, and ρ_{B} are all functions of λ. The absorption coefficient of Si is calculated using the widely used semi-empirical model [18,19]. For GaAs, the absorption coefficient was calculated using the combined Urbach-edge model [20] and the empirical high-energy absorption coefficient model [8]. The front surface reflectivity is obtained from experimental data of a graded-index anti-reflective coating [22]. The back-surface reflectivity is calculated using the Fresnel equations.

In solving the abovementioned continuity equation, the following boundary conditions are used:

$$D_{\mathrm{n}} \frac{dn}{dx}\Big|_{x=0} = S_{\mathrm{n,F}}[\delta n(0)] \qquad (13.a)$$

and
$$D_{\mathrm{n}} \frac{dn}{dx}\Big|_{x=L} = -S_{\mathrm{n,B}}[\delta n(L)] - J_{\mathrm{net}} / e \qquad (13.b)$$

, where $S_{\mathrm{n,F}}$ and $S_{\mathrm{n,B}}$ are the surface recombination velocities at the front and back surfaces, respectively and $\delta n(x) = n(x) - n_{\mathrm{eq}}$. These boundary conditions allow the modelling of electric contacts as well as the back-surface field (BSF) effect if the corresponding effective surface recombination velocities are used. However, a more rigorous analysis of the BSF can be performed using the drift-diffusion approach as discussed above. The hole concentration under

the charge-neutrality approximation can be obtained as $p(x) = \delta n(x) + p_{\text{eq}}$. The recombination velocities at the front and back surfaces were taken from [8]. The non-equilibrium radiative recombination rate is calculated as

$$R_{\text{Radiative}} = \frac{2\pi}{h^3 c^2 L} \int_{E_g}^{\infty} \varepsilon_{\text{em}}(\omega) \; \frac{(\hbar\omega)^2}{\exp(\hbar\omega / k_B T_E) - 1} \; d(\hbar\omega) \; \left(\frac{np}{n_{\text{eq}} p_{\text{eq}}} - 1\right) \quad (14)$$

, where $\hbar$ is the reduced Planck's constant, ω is the angular frequency, c is the speed of light, L is the thickness of the emitter, and $\varepsilon_{\text{em}}(\omega)$ is the spectral emissivity of the radiating surface, which was calculated based on the photon absorption profile inside the semiconductor.

The Auger recombination rate is given by

$$R_{\text{Auger}} = C_{\text{n}}(n^2 p - n_{\text{eq}}^2 p_{\text{eq}}) + C_{\text{p}}(np^2 - n_{\text{eq}} p_{\text{eq}}^2) \quad (15)$$

, where C_{n} and C_{p} are the electron and hole Auger recombination coefficients, respectively. The Auger recombination coefficients for Si and GaAs were taken from [8,12]. The Shockley-Reed-Hall (SRH) recombination rate is

$$R_{\text{SRH}} = \frac{np - n_{\text{i}}^2}{\tau_{\text{P}}(n + n_1) + \tau_{\text{n}}(p + p_1)} \quad (16)$$

, where $n_1 = n_{\text{eq}} \exp[(E_{\text{T}} - E_{\text{f}}) / k_{\text{B}} T_{\text{E}}]$ and $p_1 = p_{\text{eq}} \exp[(E_{\text{f}} - E_{\text{T}}) / k_{\text{B}} T_{\text{E}}]$. In the above equations, E_{f} is the Fermi level and E_{T} is the trap energy level, which we considered to be at the intrinsic energy level. τ_{n} and τ_{P} are the SRH recombination lifetimes for electrons and holes, respectively. The above-mentioned detailed recombination models are valid under both low and high injection levels. The values of the SRH recombination lifetimes in Si and GaAs are taken from available literature [13,14].

Supplementary Note 2. Phase space analysis of the thermionically emitted electrons in the space charge mode of a thermionic energy converter

The space charge limited regime in a thermionic energy converter (TEC) is characterized by two boundary points. The onset of the space charge limited regime, where φ_{m} (maximum motive in the interelectrode space) takes place at the point just outside the collector (see Fig. 1(b) of the main article), is known as the critical point. This point is distinguished by J_{ER} and

V_C as the critical current density and applied voltage, respectively. On the other hand, when φ_m coincides with the point just outside the emitter, the device is at the saturation point. This point is identified by J_{ES} and V_S. To calculate the characteristics in the space charge limited mode, the electron velocity distribution function, $f(x,v_x)$, the electron number density in the interelectrode space, $n(x)$, and the dimensionless Poisson's equations need to be obtained and solved, where x and v_x are the position in the interelectrode space and electron velocity, respectively. In the case of collisionless electrons, Vlasov's equation can be solved analytically to determine the velocity distribution function of electrons. This analytical solution results in a Hemi-Maxwellian electron velocity distribution at the position of maximum motive, x_m ; *i.e.*, for $v_x > 0$, the distribution is Maxwellian, whereas for $v_x < 0$, the distribution is zero. The velocity distribution at the maximum motive position, $f(x_M, v_x)$, can be used as a boundary condition to calculate the electron velocity distribution in other positions. Within the region $x > x_m$, the electrons are accelerated by the negative space charge since the gradient of the motive is always negative. Consequently, these electrons undergo a motive difference of $(\varphi_m - \varphi_x)$ corresponding to $v_{x,\min} = \sqrt{2(\varphi_m - \varphi_x)/m_e}$, where m_e is the mass of an electron. Therefore, the velocity distribution of the electrons which could overcome the maximum motive barrier can be written as

$$f(x,v_x) = 2n(x_m)\sqrt{m_e^3/(8\pi^3 k_B^3 T_E^3)}\exp\left(\frac{\varphi_m - \varphi_x - \frac{1}{2}m_e v_x^2}{k_B T_E}\right)\Theta(v_x - v_{x,\min}) \quad (17)$$

, where k_B is the Boltzmann constant, T_E is the emitter temperature, $v = \sqrt{v_x^2 + v_y^2 + v_z^2}$ is the electron velocity, and Θ is the Heaviside step function.

On the other hand, for $x < x_m$, the electrons are decelerated by the electric field and can have both

positive and negative velocities along the x direction. This leads to a negative minimum velocity along the x direction, $v_{x,\min} = -\sqrt{2(\varphi_m - \varphi_x)/m_e}$ .Therefore, when $x \le x_m$, the velocity distribution function is given by

$$f(x,v_x)=2n(x_{\mathrm{m}})\sqrt{m_{\mathrm{e}}^3/(8\pi^3k_{\mathrm{B}}^3T_{\mathrm{E}}^3)}\exp\left(\frac{\varphi_{\mathrm{m}}-\varphi_x-\frac{1}{2}m_{\mathrm{e}}v_x^2}{k_{\mathrm{B}}T_{\mathrm{E}}}\right)\Theta(v_x+|v_{x,\min}|).\quad(18)$$

Consequently, electrons on either side of x_{m} also undertake a Hemi-Maxwellian velocity distribution with the peak shifted from 0 to $v_{x,\min}$. By integrating the velocity distribution function over the entire velocity space at x, one can find the spatial profile of the electron density as

$$n(x)=\int_{-\infty}^{+\infty}dv_z\int_{-\infty}^{+\infty}dv_y\int_{-\infty}^{+\infty}dv_x f(x,v)=n(x_{\mathrm{m}})\exp(\gamma)[1\pm\mathrm{erf}(\gamma)]\quad(19)$$

, where $\gamma=(\varphi_{\mathrm{m}}-\varphi(x))/k_{\mathrm{B}}T_{\mathrm{E}}$ is the dimensionless potential barrier and $\mathrm{erf}(z)=\frac{2}{\sqrt{\pi}}\int_0^z\exp(-t^2)dt$ is the error function [23,24]. In Supplementary Equation (19), the upper sign is for $x\le x_{\mathrm{m}}$ and the lower sign is for $x>x_{\mathrm{m}}$. Now, the potential barrier profile (see Fig. 1(b) of the main article), $\varphi(x)$, arising from the mutual repulsion of the electrons in the interelectrode space, can be obtained from the Poisson equation, $\frac{d^2\varphi}{dx^2}=-e^2n(x)/\varepsilon_0$, where ε_0 is the permittivity of free space. By substituting the electron distribution expression from Supplementary Equation (19) into the Poisson equation and dividing the position variable by the Debye length, $L_D=\sqrt{\varepsilon_0k_{\mathrm{B}}T_{\mathrm{E}}/2n(x_{\mathrm{m}})e^2}$, where e is the electron charge, we obtain the dimensionless Poisson equation

$$\frac{d^2\gamma}{d\xi^2}=\exp(\gamma)[1\pm\mathrm{erf}(\sqrt{\gamma})]\quad(20)$$

, where $\xi=(x-x_{\mathrm{m}})/L_{\mathrm{D}}$. The '-' sign applies for $\xi<0$ and the '+' sign is for $\xi\ge0$.

Double integrating the Supplementary Equation (20) with appropriate boundary conditions leads to

$$\xi=-\int_0^{\gamma}\frac{dt}{[\exp(t)\pm\exp(t)\mathrm{erf}(\sqrt{t})\mp2\sqrt{\frac{t}{\pi}}-1]^{\frac{1}{2}}}\quad(21)$$

, where the upper sign applies for $\xi < 0$ and the lower sign applies for $\xi \geq 0$. In arriving at the above integral solution, we have used the value of dimensionless potential barrier and its slope in the position of maximum motive in the interelectrode space as boundary conditions. We have calculated the value of this integral numerically for a wide range of γ . The value of φ_{m} depends on the operating voltage, which can be summarized for the saturation, space charge and retarding modes of operation [7,23,25] as

$$\varphi_{\mathrm{m}} = \begin{cases} \varphi_{\mathrm{E}},\ 0 < V < V_{\mathrm{S}} \\ \varphi_{\mathrm{E}} + \gamma_{\mathrm{E}} k_{\mathrm{B}} T_{\mathrm{E}},\ V_{\mathrm{S}} < V < V_{\mathrm{C}} \\ \varphi_{\mathrm{C}} + eV,\ V > V_{\mathrm{C}} \end{cases} \qquad (22)$$

, where γ_{E} is the value of γ at the emitter surface and is given by $\gamma_{\mathrm{E}} = \ln(J_{\mathrm{ES}} / J_{\mathrm{E}})$, in which J_{ES} is the emitter saturation current density and J_{E} is the emitter current density at voltage V . φ_{E} and φ_{C} are the emitter and collector work functions, respectively. V_{S} and V_{C} are the saturation and critical point voltages, respectively, and are defined as follows. When $V = V_{\mathrm{S}}$, the maximum energy barrier occurs just outside the emitter, and all electrons originating from the emitter can reach the collector. This voltage can be expressed as

$$eV_{\mathrm{S}} = \varphi_{\mathrm{E}} - \varphi_{\mathrm{C}} - \gamma_{\mathrm{C}}(\xi_{\mathrm{CS}}) k_{\mathrm{B}} T_{\mathrm{E}} \qquad (23)$$

, where γ_{C} is the value of γ at the collector surface and $\xi_{\mathrm{CS}} = 9.186 \times 10^{5} \sqrt{J_{\mathrm{ES}}} d / T_{\mathrm{E}}^{3/4}$ [24]. When $V = V_{\mathrm{C}}$, the maximum energy barrier occurs just in front of the collector and all the electrons originating from the emitter need to overcome a decelerating force. The critical point voltage V_{C} can be defined as

$$eV_{\mathrm{C}} = k_{\mathrm{B}} T_{\mathrm{E}} \ln(A_{\mathrm{R}} T_{\mathrm{E}}^{2} / J_{\mathrm{ER}}) - \varphi_{\mathrm{C}} \qquad (24)$$

, where J_{ER} is the emitter current density at the critical point [24], which is calculated precisely in the present study using the method described in [25]. A_{R} is the Richardson constant. For $V < V_{\mathrm{S}}$, the emitter current density is the saturation current density, $J_{\mathrm{ES}} = A_{\mathrm{R}} T_{\mathrm{E}}^{2} \exp[-(\varphi_{\mathrm{E}} - E_{\mathrm{F,n}} + E_{\mathrm{F,E}}) / k_{\mathrm{B}} T_{\mathrm{E}}]$, and for $V > V_{\mathrm{C}}$, it is given by $J_{\mathrm{E}} = \frac{n(L)}{n_{\mathrm{eq}}} A_{\mathrm{R}} T_{\mathrm{E}}^{2} \exp[-(\varphi_{\mathrm{C}} + eV) / k_{\mathrm{B}} T_{\mathrm{E}}]$ and the device is in the retarding mode. Note that Maxwell-Boltzmann statistics are assumed in deriving the above-mentioned current equations as, at the energy range relevant to thermionic emission, Maxwell-Boltzmann and Fermi-Dirac statistics become virtually indistinguishable. For $V_{\mathrm{S}}<V<V_{\mathrm{C}}$, the device is in the space charge regime and the emitter current can be calculated using the method described in [25], which we briefly review here. For a given φ_{E} and T_{E}, we first determine the emitter current density at the saturation point (J_{ES}) and critical point (J_{ER}). Then, for a particular current density (J_{E}) in the space charge region $(J_{\mathrm{ER}} < J_{\mathrm{E}} < J_{\mathrm{ES}})$, we calculate γ_{E}. Using this value of γ_{E}, we then calculate ξ_{E}, then ξ_{C} and finally γ_{C} using the curve resulting from Supplementary Equation (21). The voltage in the space charge region can then be calculated as $eV = \varphi_{\mathrm{E}} - \varphi_{\mathrm{C}} + (\gamma_{\mathrm{E}} - \gamma_{\mathrm{C}}) k_{\mathrm{B}} T_{\mathrm{E}}$. For the numerical implementation of this method, we follow the algorithm described in [25].

The reverse current from the collector in the saturation, space charge and retarding regions can be defined as

$$J_{\mathrm{C}} = A_{\mathrm{R}} T_{\mathrm{C}}^{2} \exp[-(\varphi_{\mathrm{m}} - eV) / k_{\mathrm{B}} T_{\mathrm{C}}]. \qquad (25)$$

In the above equation, we neglected the quasi-Fermi level splitting in the n-type collector because, in the emitter temperature ranges under consideration, the thermal radiation is dominated by sub-bandgap photons. Moreover, even if we consider the electron-hole pairs

generated due to that small portion of above-bandgap photons, these excess carriers will recombine through various processes and their steady-state contribution to the electron quasi-Fermi level splitting will be vanishingly small. This is because the collector is heavily n-doped, and hence it already contains a large electron concentration from dopant ionization.

Using the above analysis, the net energy flux carried by the thermionic current from the emitter is given by

$$Q_{\mathrm{T}} = \frac{[(J_{\mathrm{E}} - J_{\mathrm{C}})\varphi_{\mathrm{m}} + 2k_{\mathrm{B}}(T_{\mathrm{E}}J_{\mathrm{E}} - T_{\mathrm{C}}J_{\mathrm{C}})]}{e}. \qquad (26)$$

The first term in Supplementary Equation (26), is due to the potential energy and the second term is due to the average thermal energy [26]. The net current density can be written as

$$J_{\mathrm{net}} = J_{\mathrm{E}} - J_{\mathrm{C}} \,. \qquad (27)$$

Supplementary Note 3. The interelectrode thermal radiation model

The interelectrode thermal radiation loss in a PETE device has a strong dependence on the gap width value and, therefore, should be properly calculated using a physically consistent model. This is because the optical properties of the electrode materials in a PETE device depend on the doping concentration and temperature. Moreover, when the interelectrode distance is on the order of a few micrometers, photon tunneling (due to the coupling of the evanescent waves) can cause a substantial increase in radiative exchange, a phenomenon also known as the near-field effect of thermal radiation. To address these concerns, in this work, we have taken an *ab initio* approach to calculate the thermal radiation loss using fluctuational electrodynamics [27,28]. This approach considers the near-field coupling of thermal radiation and reduces to the Stefan-Boltzmann law in the far-field limit, thereby making it independent of any assumption regarding the transition between these two regimes. To illustrate this model, let us consider the generic case of radiative heat transfer between two semi-infinite media which are separated by a vacuum gap as shown in Supplementary Fig. 6(a). The two media are each at a thermal equilibrium but have different temperatures. According to the fluctuation-dissipation theorem [28], thermal radiation in a medium is originated from the random movement of charges or dipoles inside that medium at finite temperature. The random thermal fluctuations produce a space-time dependent

electric current density $\mathbf{j}(\mathbf{x},t)$ inside the medium whose time average is zero. This fluctuating current will result in a fluctuating electromagnetic field. As shown in Supplementary Fig. 6(b), the electromagnetic field at any location is a superposition of contributions from all point sources in the radiating medium. The electromagnetic waves deep inside the medium will be attenuated due to absorption inside the medium.

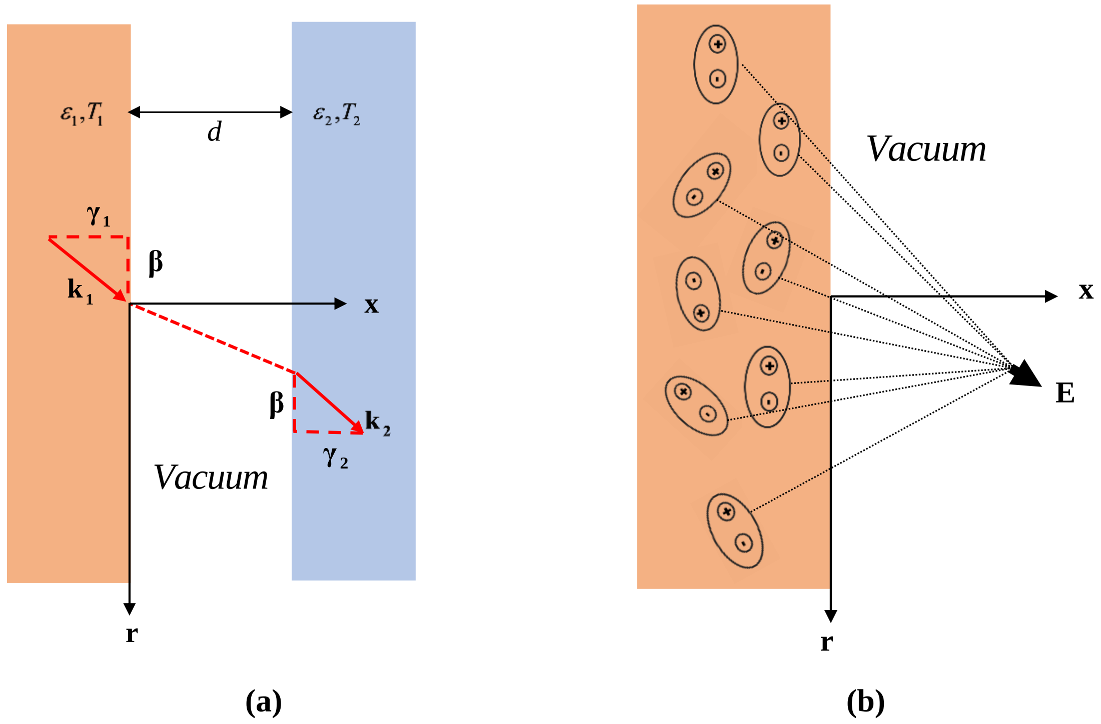

Supplementary Fig. 6. Illustration of the interelectrode thermal radiation in a TEC. (a) Radiative coupling between two parallel plates separated by a vacuum gap of thickness *d*, and (b) the electric field near a radiating surface due to thermal fluctuation of the charge dipoles. The axes are shown in a cylindrical coordinate system where the x-direction is perpendicular to the interface between the radiating surface and vacuum. The wavevectors $\mathbf{k}_1$, $\mathbf{k}_2$ are composed of components that are parallel (**β**) and perpendicular (γ_1, γ_2) to the interfaces, respectively.

For a point source at a position $\mathbf{x}$ inside the medium, the resulting electric field at a point $\mathbf{x}'$ near the medium can be expressed in the Fourier domain as [28]

$$\mathbf{E}(\mathbf{x}',\omega) = i\,\omega\mu_0 \int_V \overline{\overline{\mathbf{G}}}(\mathbf{x},\mathbf{x}',\ \omega).\ \mathbf{j}(\mathbf{x},\omega)\mathbf{dx} \qquad (28)$$

, where μ_0 is the magnetic permeability of the free space and the volume integral is over the region that contains the fluctuating sources. $\overline{\overline{\mathbf{G}}}(\mathbf{x},\mathbf{x}',\omega)$ is a spatial transfer function (also known as the

dyadic Green's function) [28], which relates the current source ($\mathbf{j}$) at $\mathbf{x}$ to the resultant electric field ($\mathbf{E}$) at $\mathbf{x'}$ and satisfies the vector Helmholtz equation:

$$\nabla \times \nabla \times \overline{\overline{\mathbf{G}}}(\mathbf{x}, \mathbf{x'}, \omega) - k^2 \overline{\overline{\mathbf{G}}}(\mathbf{x}, \mathbf{x'}, \omega) = \overline{\overline{\mathbf{I}}}\delta(\mathbf{x} - \mathbf{x'}) \qquad (29)$$

, where k is the amplitude of the wavevector at $\mathbf{x'}$, and $\overline{\overline{\mathbf{I}}}$ is a unit dyadic. The corresponding magnetic field $\mathbf{H}(\mathbf{x'}, \omega)$ can be obtained from the Maxwell equation as

$$\mathbf{H}(\mathbf{x'}, \omega) = \frac{1}{\mathrm{i}\omega\mu_0} \nabla \times \mathbf{E}(\mathbf{x'}, \omega). \qquad (30)$$

These electromagnetic waves can either propagate along the free space or decay evanescently depending on their angle of incidence at the interface between the radiating medium and the vacuum space. The energy flux carried by these electromagnetic waves can be calculated from the ensemble average of the Poynting vector as

$$\langle \mathbf{S}(\mathbf{x'}, \omega) \rangle = \frac{1}{2} \langle \mathrm{Re}[\mathbf{E}(\mathbf{x'}, \omega) \times \mathbf{H}^*(\mathbf{x'}, \omega)] \rangle \ . \qquad (31)$$

If a second medium is brought close enough to the radiating medium, these electromagnetic waves will be absorbed by that second medium and the resulting electronic motion will dissipate the energy in the form of heat. Depending on the distance between the two media, either propagating waves (when the distance is large) or both propagating and evanescent waves (when the distance is of the order of the characteristic wavelength of thermal radiation given by Wien's displacement law) can be coupled to the second medium. The former mechanism is governed by the Stefan- Boltzmann law while the latter may result in a super Planckian radiative exchange due to coupling of the evanescent waves, also known as radiation tunneling. Similarly, the first medium will absorb any thermal radiation which is emitted by the second medium resulting in a net radiative exchange between the two media.

The above discussion provides a general treatment of modelling thermal radiation and its dissipation using the *ab initio* approach. For the numerical implementation of this model, we have used the dyadic Green's functions and the scattering matrix method [29]. The computational details will not be repeated here for the sake of brevity.

Supplementary Note 4. Dielectric permittivity models

I. Silicon

Silicon is an indirect bandgap semiconductor and the fundamental absorption (interband absorption) occurs at wavelengths shorter than λ_{g}, which corresponds to the band-gap energy E_{g}. Free carrier absorption can also contribute to dielectric properties for doped silicon where the impurities are ionized to generate free carriers. In this work, we have modelled the silicon dielectric permittivity with a Drude model considering the various absorption mechanisms such as interband transition, lattice vibration, and intraband free carrier absorption. The resulting complex dielectric function, which is related to the refractive index (n) and the extinction coefficient (κ), can be written as [30]

$$\varepsilon(\omega) = \varepsilon_{\mathrm{bl}} - \frac{N_{\mathrm{e}} e^2 / \varepsilon_0 m_{\mathrm{e}}^*}{\omega^2 + \mathrm{i}\omega / \tau_{\mathrm{e}}} - \frac{N_{\mathrm{h}} e^2 / \varepsilon_0 m_{\mathrm{h}}^*}{\omega^2 + \mathrm{i}\omega / \tau_{\mathrm{h}}}. \qquad (32)$$

In the above equation, $\varepsilon_{\mathrm{bl}}$ accounts for contributions by transitions across the bandgap and lattice vibrations, the second term is for transitions in the conduction band (free electrons), and the last term is the Drude term for transitions in the valence band (free holes). N_{e} and N_{h} are the free carrier concentrations, m_{e}^* and m_{h}^* are the effective masses, and τ_{e}, τ_{h} are the scattering times of free electrons and holes, respectively. The effective mass values are taken from [31] as $m_{\mathrm{e}}^* = 0.27 m_0$ and $m_{\mathrm{h}}^* = 0.37 m_0$, where m_0 is the electron mass in vacuum.

The scattering times τ_{e} and τ_{h} depend on the collisions of electrons and holes with lattice (phonons) and ionized dopant sites (impurities or defects). The total scattering time, for the case of τ_e, can be calculated by Matthiessen's rule:

$$\frac{1}{\tau_{\mathrm{e}}} = \frac{1}{\tau_{\mathrm{el}}} + \frac{1}{\tau_{\mathrm{ed}}} \qquad (33)$$

, where τ_{el} and τ_{ed} denote the electron–lattice and electron–defect scattering times, respectively. Similarly, τ_{h} can be related to τ_{hl} and τ_{hd}. Modelling the temperature dependence of the scattering time is more complicated. Theory predicted that the carrier-impurity scattering times vary with $T^{1.5}$ and the carrier-lattice scattering times due to acoustic phonons vary with $T^{-1.5}$

[15]. As the temperature increases, the scattering rate due to impurity tends to decrease because the electrostatic force that governs dopant sites becomes weaker and carriers can move more agilely. On the other hand, the carrier-lattice scattering rate increases with the temperature due to the increased phonon number density (occupation number). Therefore, lattice scattering dominates the scattering process at high temperatures even for heavily doped silicon. The temperature dependence of the electron-defect scattering in Si can be written as [30]

$$\frac{\tau_{\mathrm{ed}}}{\tau^0_{\mathrm{ed}}} = \frac{\tau_{\mathrm{hd}}}{\tau^0_{\mathrm{hd}}} = \left(\frac{T}{300}\right)^{1.5} . \qquad (34)$$

On the other hand, the temperature dependence of the lattice scattering time is obtained from an empirical fitting which takes into account the contribution from both optical and acoustic phonon modes and can be written as [30]

$$\frac{\tau_{\mathrm{el}}}{\tau^0_{\mathrm{el}}} = \left(\frac{T}{300}\right)^{-3.8} \text{ and } \frac{\tau_{\mathrm{hl}}}{\tau^0_{\mathrm{hl}}} = \left(\frac{T}{300}\right)^{-3.6} . \qquad (35)$$

In addition to temperature, the doping dependences of these scattering times are also taken from experimental fitting as [32]

$$\tau^0_{\mathrm{e}} = \frac{19.5}{1+(N_{\mathrm{D}}/1.3\times10^{17})^{0.91}} + 141 \qquad (36)$$

and

$$\tau^0_{\mathrm{h}} = \frac{94}{1+(N_{\mathrm{A}}/1.9\times10^{17})^{0.76}} + 10 \qquad (37)$$

, where superscript 0 denotes values at 300 K, the scattering time is in fs (10^{-15} s), and $N_{\mathrm{D}}(N_{\mathrm{A}})$ is the n-type (p-type) dopant concentration and is in cm^{-3}.

The lattice contribution can be obtained from the room temperature lattice mobilities of 1451 $\mathrm{cm}^2\ \mathrm{V}^{-1}\mathrm{s}^{-1}$ for electrons and 502 $\mathrm{cm}^2\ \mathrm{V}^{-1}\mathrm{s}^{-1}$ for holes [33]. Therefore, $\tau^0_{\mathrm{el}} = 2.23 \times 10^{-13}\,\mathrm{s}$ and $\tau^0_{\mathrm{hl}} = 1.06 \times 10^{-13}\,\mathrm{s}$. The values of τ^0_{ed} and τ^0_{hd} can be solved by combining Supplementary Equations 33, 36 and 37.

The carrier concentrations N_e and N_h in Supplementary Equation 32 depend on temperature and dopant concentrations. In the case of an n-doped semiconductor, these carrier concentrations can be obtained as [15,34]

$$N_e = \frac{1}{2}[N_D + \sqrt{N_D^2 + 4N_i^2}] \, . \qquad (38)$$

In the above equation, N_i is the intrinsic carrier concentration and can be written as $N_i = \sqrt{N_C N_V} \exp(-E_g / 2k_B T)$, where N_C and N_V are the effective densities of states in the conduction band and valance band, respectively. The corresponding hole concentration can be written as $N_h = N_i^2 / N_e$. On the other hand, for a p-doped semiconductor, the carrier concentrations follow:

$$N_h = \frac{1}{2}[N_A + \sqrt{N_A^2 + 4N_i^2}] \ (39.a) \quad \text{and} \ N_e = N_i^2 / N_h. \ (39.b)$$

This dielectric model is fed into the fluctuational electrodynamics model of thermal radiation to calculate the interelectrode radiative exchange.

II. GaAs

For the dielectric permittivity of GaAs, we consider an empirical Lorentz-Drude model. It consists of a phonon lattice vibrational term and a free carrier plasma term and can be written as

$$\varepsilon(\omega) = \varepsilon_\infty \left(1 + \frac{\omega_{LO}^2 - \omega_{TO}^2}{\omega_{TO}^2 - \omega^2 + i\omega\Gamma} - \frac{\omega_p^2}{\omega^2 - i\omega\gamma}\right) . \qquad (40)$$

In the above equation, ε_∞, ω_{TO} (ω_{LO}), Γ, ω_p and γ are the high-frequency dielectric constant, frequency of the transverse (longitudinal) zone-center optical phonon, phonon damping constant, plasma frequency and plasma damping constant, respectively. The phonon parameters and ε_∞ in the abovementioned effective-plasmon dielectric function are taken from [35]. The plasma frequency and the plasma damping constant in the Drude term can be written as

$$\omega_p^2 = \frac{N_{d/a} \, e^2}{\varepsilon_0 \varepsilon_\infty m_{e/h}^*} \ (41.a) \quad \text{and} \ \gamma = \frac{e}{m_{e/h}^* \, \mu_{e/h}\left(N_{d/a}, T\right)} \ . \ (41.b)$$

In the above equation, $N_{\mathrm{d/a}}$ is the doping concentration (electron concentration N_{d} or hole concentration N_{a}), e is the electron charge, $m^*_{\mathrm{e/h}}$ is the electron or hole density of states effective mass, $\mu_{\mathrm{e/h}}$ is the mobility of electrons or holes (taken from an empirical low-field mobility model [17]), and ε_0 is the permittivity of free space.

Supplementary Note 5. Semiconductor material model

The equilibrium Fermi level of the emitter can be calculated from the charge neutrality criterion in the semiconductor crystal, which for a p-type semiconductor can be written as [36]

$$N_{\mathrm{C}} \exp[-(E_{\mathrm{g}} - E_{\mathrm{f}}) / k_{\mathrm{B}} T_{\mathrm{E}}] + N_{\mathrm{A}} \frac{1}{1 + 4\ \exp[(E_{\mathrm{A}} - E_{\mathrm{f}}) / k_{\mathrm{B}} T_{\mathrm{E}}]} = N_{\mathrm{V}} \exp(-E_{\mathrm{f}} / k_{\mathrm{B}} T_{\mathrm{E}}). \quad (42)$$

In the above equation, N_{C} and N_{V} are the effective densities of states in the conduction and valance band, respectively, E_{g} is the band-gap of the semiconductor, and E_{A} is the dopant energy level for which we considered boron impurities in Si [12] and carbon impurities in GaAs [12]. The temperature dependences of the effective densities of states and bandgap are given by

$$N_{\mathrm{C}} = 2\left(\frac{2\pi m^*_{\mathrm{n}} k_{\mathrm{B}} T}{h^2}\right)^{3/2}, \quad (43.\mathrm{a})$$

$$N_{\mathrm{V}} = 2\left(\frac{2\pi m^*_{\mathrm{p}} k_{\mathrm{B}} T}{h^2}\right)^{3/2}, \quad (43.\mathrm{b})$$

$$\text{and } E_{\mathrm{g}}(T) = E_{\mathrm{g0}} - aT^2 / (T + b). \quad (43.\mathrm{c})$$

In the above equations, m^*_{n} and m^*_{p} are the density of states effective masses of the semiconductor; E_{g0} is the material bandgap at 0 K, and *a,b* are the empirical fitting parameters of the bandgap narrowing effect, which are taken from [12] for Si and GaAs, respectively. Once the equilibrium Fermi level is determined, the equilibrium electron and hole concentration can be calculated as follows:

$$n_{\mathrm{eq}} = N_{\mathrm{C}} \exp[-(E_{\mathrm{g}} - E_{\mathrm{f}}) / k_{\mathrm{B}} T_{\mathrm{E}}] \quad (44.\mathrm{a}) \text{ and } \quad p_{\mathrm{eq}} = N_{\mathrm{V}} \exp(-E_{\mathrm{f}} / k_{\mathrm{B}} T_{\mathrm{E}}). \quad (44.\mathrm{b})$$

Similarly, the emitter work function is related to the Fermi level as

$$\varphi_{\mathrm{E}} = E_{\mathrm{g}} - E_{\mathrm{f}} + \chi \quad (45)$$

, where χ is the electron affinity of the emitter. The diffusion coefficient of electrons D_{n} is calculated using the general relationship

$$D_{\mathrm{n}} = \frac{k_{\mathrm{B}} T_{\mathrm{E}} \mu_{\mathrm{n}}}{e} F_{1/2}\left(\frac{E_{\mathrm{f}} - E_{\mathrm{C}}}{k_{\mathrm{B}} T_{\mathrm{E}}}\right) / F_{-1/2}\left(\frac{E_{\mathrm{f}} - E_{\mathrm{C}}}{k_{\mathrm{B}} T_{\mathrm{E}}}\right) \quad (46)$$

, where μ_{n} is the electron mobility whose temperature and doping dependences were considered using a semi-empirical model [16] for Si and a low-field empirical model for GaAs [17]. F_n is the Fermi-Dirac integral of order *n*. Under low injection level, the above equation reduces to Einstein's relation.

Supplementary Note 6. Electrode thermal balance and the self-consistent iterative models

Since the thermionic solar cell's performance strongly depends on the electrode temperatures, it is crucial to calculate these temperatures and their dependence on the incident radiation flux, the device material properties, and the operating conditions. Moreover, the transition from the photon-enhanced to the thermally enhanced mode of operation in a semiconductor thermionic solar cell is closely related to the emitter temperature. The emitter thermal balance is calculated considering various mechanisms by which heat is taken away from the emitter by electrons and photons. This can be written as

$$Q_{\mathrm{In}} = Q_{\mathrm{Recomb}} + Q_{\mathrm{Rad(Far\text{-}field\ Ambient+Interelectrode)}} + Q_{\mathrm{T}} + Q_{\mathrm{Lead(Thermal\ Conduction+Joule\ Heating)}} \ . \quad (47)$$

In the above equation, Q_{In} is the solar energy absorbed by the emitter, Q_{Recomb} is the radiative loss due to non-equilibrium radiative recombination process, $Q_{\mathrm{Rad(Far\text{-}field\ Ambient+Interelectrode)}}$ is the radiative loss to the ambient and collector electrode, Q_{T} is the heat flux taken away from the emitter by thermionic electron emission, and $Q_{\mathrm{Lead(Thermal\ Conduction+Joule\ Heating)}}$ is the heat loss in the lead due to thermal conduction and Joule heating. At this point, it should be noted that the value of the lead resistance needs to be chosen carefully to reduce the lead-related loss. This is because a very low lead resistance would reduce the lead voltage drop but increase the thermal conduction loss (due to Wiedemann–Franz law). On the other hand, a very high lead resistance would limit thermal conduction loss but again result in significant Joule heating and voltage

drop in the leads. Considering these conflicting requirements, we optimized the lead resistance value for maximum conversion efficiency as follows:

$$\eta_{\text{Lead optimized}} = \frac{SJ_{\text{net}}(V - SJ_{\text{net}}R_{\text{Lead}})_{\max}}{SQ_{\text{In}}}. \quad (48)$$

In the above equation, S is the cross-sectional area of the electrodes, R_{Lead} is the lead resistance and J_{net} is the net current density. In addition, the loss due to the voltage drop across the emitter needs to be considered when the drift-diffusion approach is employed. On the other hand, the heat flux which is absorbed by the collector and released to a heat sinking mechanism is given by

$$Q_{\text{Sink}} = K_{\text{L}}(T_{\text{C}} - T_0) = Q_{\text{Rad(Interelectrode)}} + Q_{\text{T}} + Q_{\text{Lead(Thermal Conduction+Joule Heating)}} + Q_{\text{Unabsorbed}} - P_{\text{TEC}}. \quad (49)$$

In the above equation, K_{L} is the heat transfer coefficient of the cooling fluid, T_{C} and T_0 are the collector and ambient temperatures, respectively and P_{TEC} is the useful electrical output of the thermionic device. These thermal balance conditions, in addition to the coupling between the emitter particle balance and the space charge effect, were implemented in our model using a self-consistent iterative algorithm as shown in Supplementary Fig. 7.

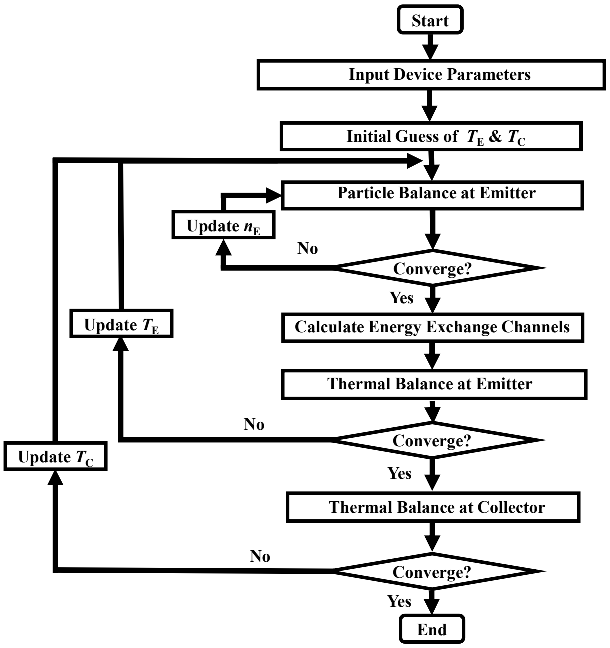

Supplementary Fig. 7. A flowchart of the self-consistent numerical iterative algorithm implemented in this work for solving the device operation. The particle balance algorithm is shown for the zero-field (in the emitter) approach.

The self-consistent iterative algorithm to solve the emitter particle balance using the drift-diffusion approach is shown in Supplementary Fig. 8.

For the convergence criterion, we used a relative tolerance value of 0.001 for the emitter thermal balance. For the emitter particle balance, we used a relative tolerance value of 0.0001. On the other hand, a relative tolerance value of 0.001 was used for the collector thermal balance. These tolerance values have been based on the consistency of the findings with finer convergence tolerance values and hence can be justified for saving on additional computational expenses.

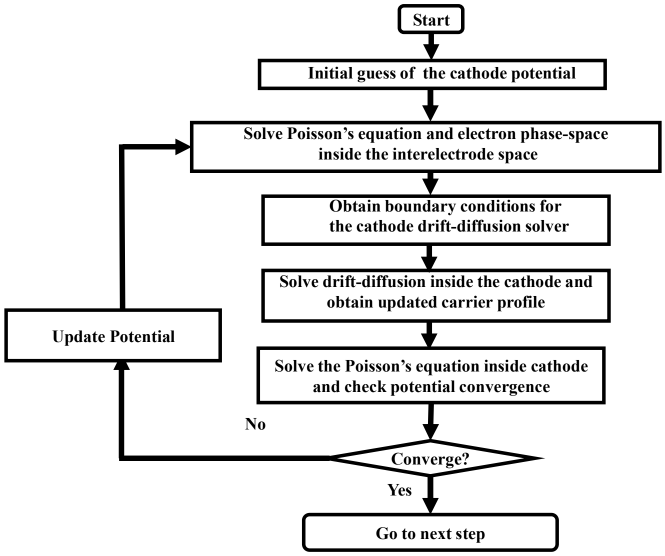

Supplementary Fig. 8. A flowchart of the self-consistent numerical iterative algorithm for solving the coupled drift-diffusion and Poisson's equations.

Supplementary Model Verifications

In this section, we validate the functionalities of the proposed self-consistent computational framework by benchmarking it against the findings reported in the existing literature. Note that for verification purposes, we had to simplify the complex interaction of various physics (which are already built into our model) to different extents to show the model's consistency with previous literature (due to the simplifications made in those works as shown in Supplementary Table 1).

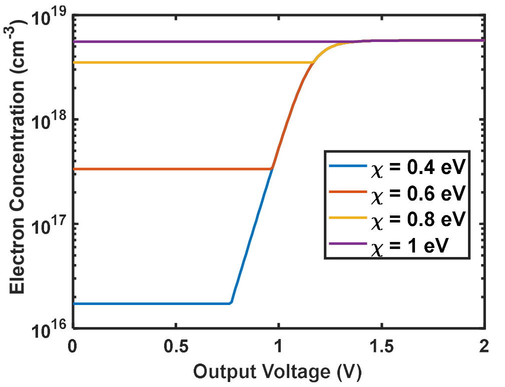

(a)

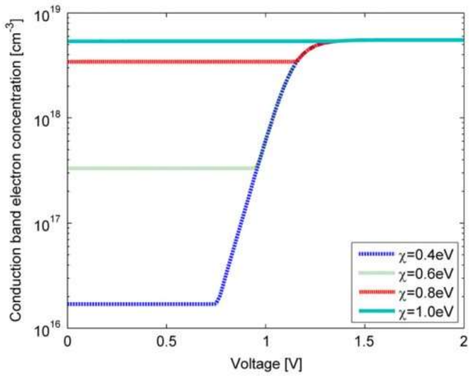

(b)

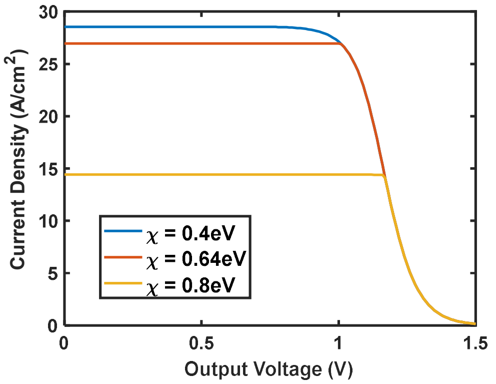

(c)

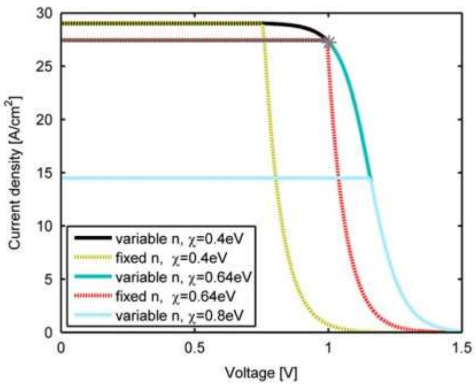

(d)

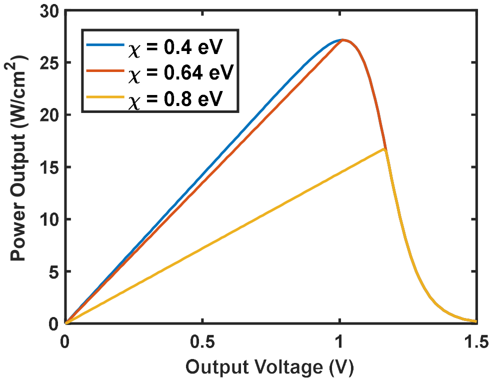

(e)

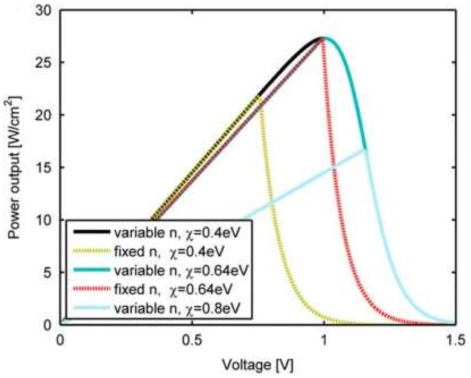

(f)

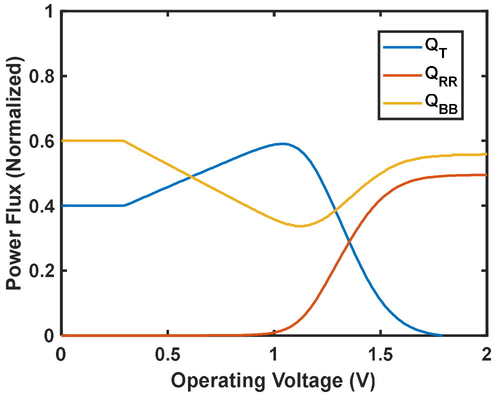

(g)

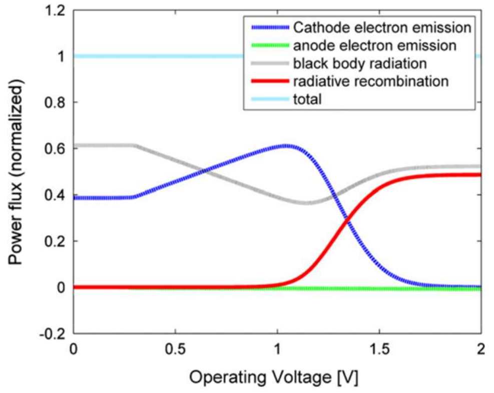

(h)

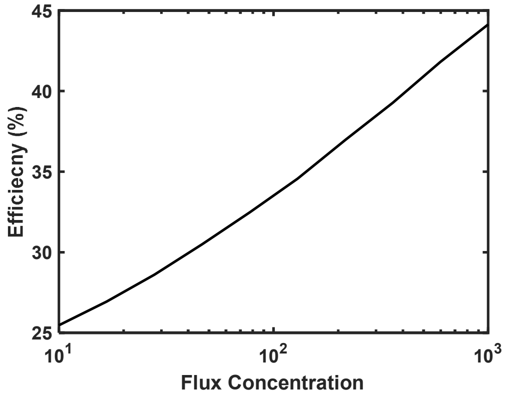

(i)

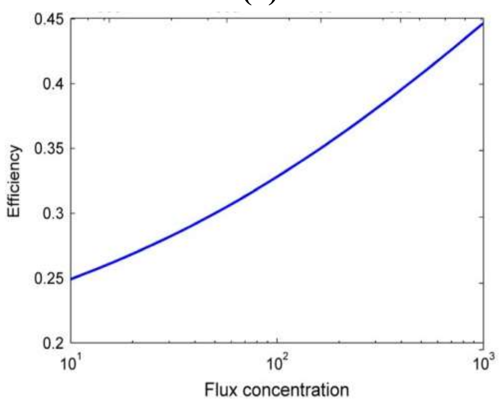

(j)

Supplementary Fig. 9. Verification of the proposed computational model with the results of Segev *et al.* [2]. [(a)-(b)] Emitter electron density as a function of the output voltage, [(c)-(d)] Current density as a function of the output voltage for different values of emitter electron affinity (the data have been benchmarked for variable electron density), [(e)-(f)] Power density as a function of the output voltage for various electron affinities of the emitter (the data have been benchmarked for variable electron density), [(g)-(h)] Various energy exchange channels as a function of the output voltage, and [(i)-(j)] Conversion efficiency as a function of the solar concentration. The figures on the left-side column represent the findings from the proposed model and the figures on the right-side column represent the data from [2] (reprinted from [2] with permission from Elsevier). Various material and device operational parameters were taken from [2] for this verification.

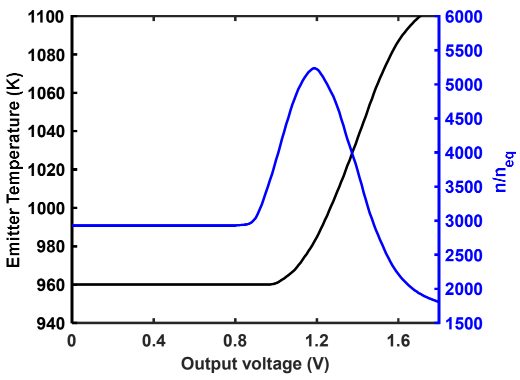

Emitter Temperature (K)
n/n_eq
Output voltage (V)

(a)

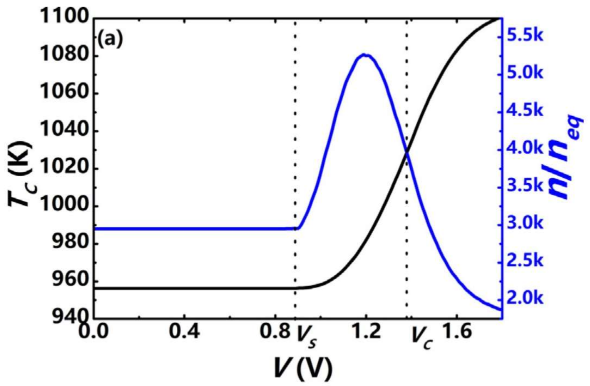

(a)
T_C (K)
n/n_eq
V_S
V_C
V (V)

(b)

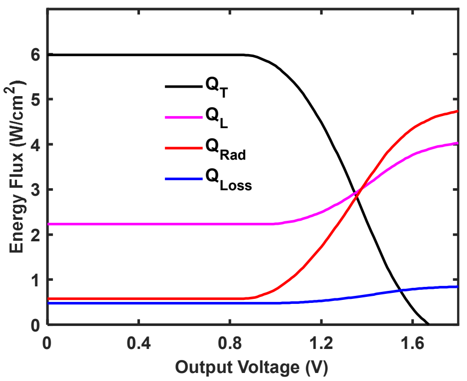

Q_T
Q_L
Q_Rad
Q_Loss
Energy Flux (W/cm^2)
Output Voltage (V)

(c)

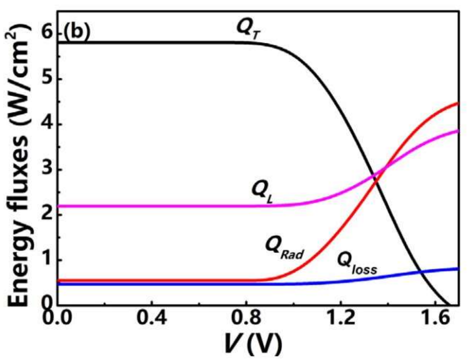

(b)
Q_T
Q_L
Q_Rad
Q_loss
Energy fluxes (W/cm^2)
V (V)

(d)

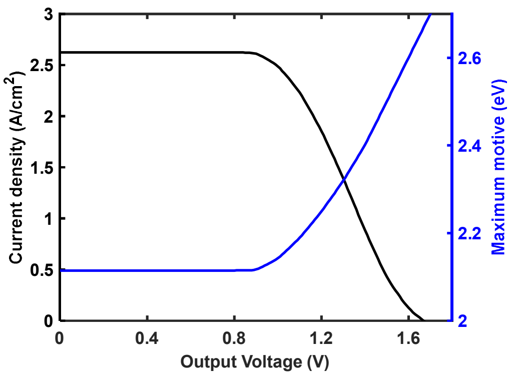

Current density (A/cm^2)
Maximum motive (eV)
Output Voltage (V)

(e)

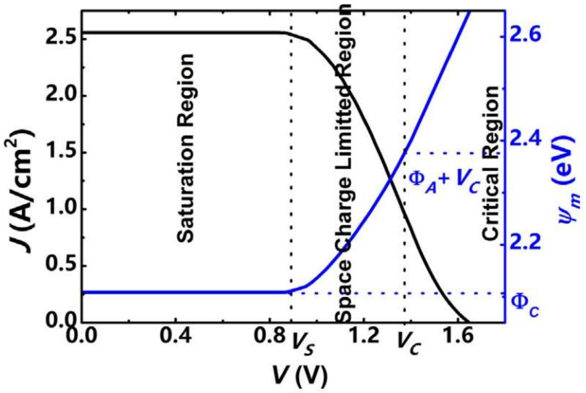

Saturation Region
Space Charge Limited Region
Critical Region
Φ_A + V_C
Φ_C
J (A/cm^2)
ψ_m (eV)
V_S
V_C
V (V)

(f)

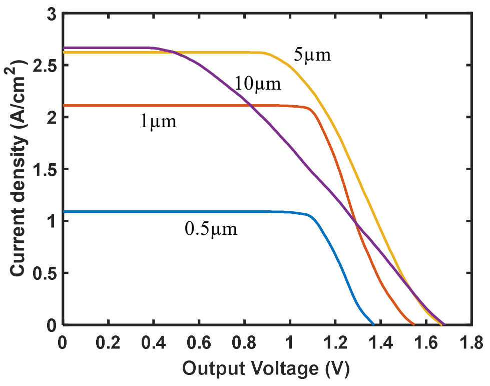

(g)

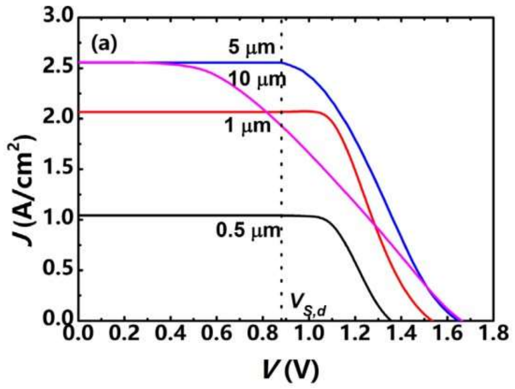

(h)

Supplementary Fig. 10. Verification of the proposed computational model with the results of Wang *et al.* [10]. [(a)-(b)] Emitter temperature and photon enhancement factor as a function of the output voltage, [(c)-(d)] Various energy fluxes as a function of the output voltage, [(e)-(f)] Current density and the maximum motive in the interelectrode space as a function of the output voltage, and [(g)-(h)] Current densities as a function of the output voltage at various interelectrode gap widths. The figures on the left-side column represent the findings from the proposed model and the figures on the right-side column represent the data reported in [10] (reprinted from [10] with permission from Elsevier). Various material and device operational parameters were taken from [10] for this verification.

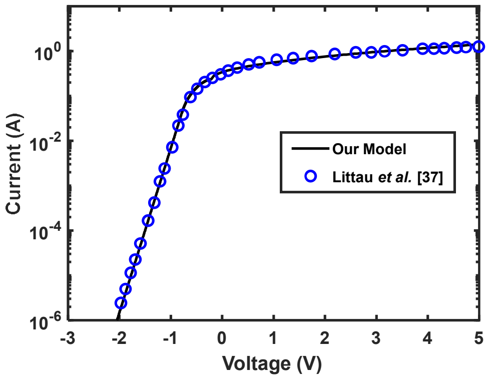

(a)

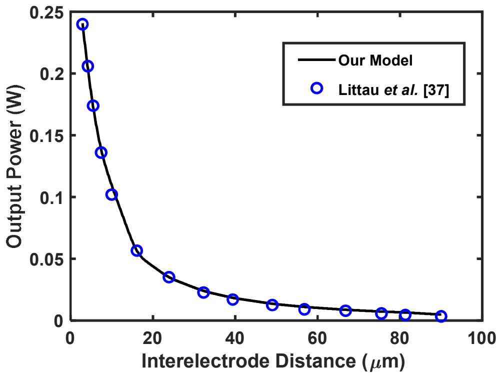

(b)

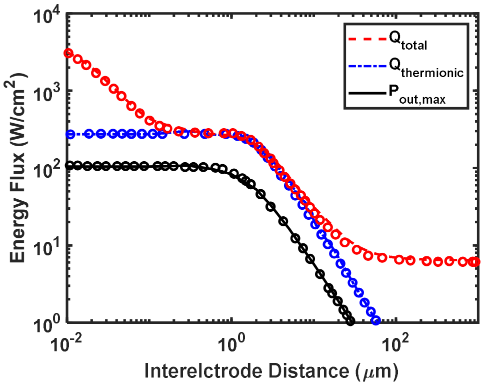

(c)

Supplementary Fig. 11. (a) The current-voltage plot produced using our model for the experimental TEC device reported in [37]. (b) Output power vs. interelectrode distance plot produced using our model for the TEC device reported in [37]. (c) Various energy fluxes vs. interelectrode distance (gap width) plots produced using our model for the TEC device reported in [38]. The curves with dash, dash-dot, and solid lines represent our results whereas the circle marks represent the data reported in [38].

Supplementary References

1. Schwede, J. W. *et al.* Photon-enhanced thermionic emission for solar concentrator systems. *Nature Materials* **9**, 762–767 (2010).

2. Segev, G., Rosenwaks, Y. & Kribus, A. Efficiency of photon enhanced thermionic emission solar converters. *Solar Energy Materials and Solar Cells* **107**, 125–130 (2012).

3. Varpula, A. & Prunnila, M. Diffusion-emission theory of photon enhanced thermionic emission solar energy harvesters. *Journal of Applied Physics* **112**, 1–5 (2012).

4. Segev, G., Rosenwaks, Y. & Kribus, A. Loss mechanisms and back surface field effect in photon enhanced thermionic emission converters. *Journal of Applied Physics* **114**, (2013).

5. Su, S., Zhang, H., Chen, X., Kang, J. & Chen, J. Parametric optimum design of a photon-enhanced thermionic solar cell. *Solar Energy Materials and Solar Cells* **117**, 219–224 (2013).

6. Sahasrabuddhe, K. *et al.* A model for emission yield from planar photocathodes based on photon-enhanced thermionic emission or negative-electron-affinity photoemission. *Journal of Applied Physics* **112**, 094907 (2012).

7. Su, S., Wang, Y., Liu, T., Su, G. & Chen, J. Space charge effects on the maximum efficiency and parametric design of a photon-enhanced thermionic solar cell. *Solar Energy Materials and Solar Cells* **121**, 137–143 (2014).

8. Varpula, A., Tappura, K. & Prunnila, M. Si, GaAs, and InP as cathode materials for photon-enhanced thermionic emission solar cells. *Solar Energy Materials and Solar Cells* **134**, 351–358 (2015).

9. Segev, G., Weisman, D., Rosenwaks, Y. & Kribus, A. Negative space charge effects in photon-enhanced thermionic emission solar converters. *Applied Physics Letters* **107**, 1–6 (2015).

10. Wang, Y., Li, H., Hao, H., Chen, J. & Su, S. Optimal design of the interelectrode space in a photon-enhanced thermionic emission solar cell. *Applied Thermal Engineering* **157**, 113758 (2019).

11. Liu, X., Xia, H. & Xuan, Y. Effects of near-field photon tunneling on the performance of photon–enhanced thermionic emission energy conversion. *Journal of Quantitative Spectroscopy and Radiative Transfer* **222–223**, 223–228 (2019).

12. Levinshtein, M. E., Rumyantsev, S. L. & Shur, M. *Handbook Series On Semiconductor Parameters* Vol. 1 (World Scientific Publishing Co. Pte. Ltd., Singapore, 1996).

13. A. Schenk. A model for the field and temperature dependence of Shockley-Read-Hall Lifetimes in silicon. *Solid-State Electronics* **35**, 1585–1596 (1992).

14. Yablonovitch, E., Bhat, R., Harbison, J. P. & Logan, R. A. Survey of defect-mediated recombination lifetimes in GaAs epilayers grown by different methods. *Applied Physics Letters* **50**, 1197–1199 (1987).

15. Sze, S. M. *Semiconductor Devices: Physics and Technology* (John Wiley & Sons, New York, 2002).

16. Reggiani, S., Valdinoci, M., Colalongo, L., Rudan, M. & Baccarani, G. Analytical, temperature-dependent model for majority- and minority-carrier mobility in silicon devices. *VLSI Design* **10**, 467–483 (2000).

17. Sotoodeh, M., Khalid, A. H. & Rezazadeh, A. A. Empirical low-field mobility model for III-V compounds applicable in device simulation codes. *Journal of Applied Physics* **87**, 2890–2900 (2000).

18. Green, M. A. *Solar cells: Operating principles, technology, and system applications* (Prentice-Hall, Hoboken, New Jersey, 1982).

19. Rajkanan, K., Singh, R. & Shewchun, J. Absorption coefficient of silicon for solar cell calculations. *Solid State Electronics* **22**, 793–795 (1979).

20. Beaudoin, M., DeVries, A. J. G., Johnson, S. R., Laman, H. & Tiedje, T. Optical absorption edge of semi-insulating GaAs and InP at high temperatures. *Applied Physics Letters* **70**, 3540–3542 (1997).

21. Incropera, F. P. & DeWitt, D. P. *Fundamentals of Heat and Mass Transfer* (John Wiley & Sons, New York, 2002).

22. Kuo, M.-L. *et al.* Realization of a near-perfect antireflection coating for silicon solar energy utilization. *Optics Letters* **33**, 2527 (2008).

23. Hatsopoulos, G. N. & Gyftopoulos, E. P. *Thermionic Energy Conversion. Vol. 1: Processes and Devices* (MIT Press, Cambridge, 1973).

24. Hatsopoulos, G. N. & Gyftopoulos, E. P. *Thermionic Energy Conversion. Vol. 2: Theory, Technology, and Application* (MIT Press, Cambridge, 1979).

25. Khoshaman, A. H. *et al.* Nanostructured Thermionics for Conversion of Light to Electricity: Simultaneous Extraction of Device Parameters. *IEEE Transactions on Nanotechnology* **14**, 624-632 (2015).

26. Lough, B. C., Lewis, R. A. & Zhang, C. Principles of charge and heat transport in thermionic devices. *Proc. SPIE, Smart Structures, Devices, and Systems II* **5649**, 332 -343 (2005).

27. Basu, S., Zhang, Z. M. & Fu, C. J. Review of near-field thermal radiation and its application to energy conversion. *International Journal of Energy Research* **33**, 1203–1232 (2009).

28. Francoeur, M. & Pinar Mengüç, M. Role of fluctuational electrodynamics in near-field radiative heat transfer. *Journal of Quantitative Spectroscopy and Radiative Transfer* **109**, 280–293 (2008).

29. Francoeur, M., Pinar Mengüç, M. & Vaillon, R. Solution of near-field thermal radiation in one-dimensional layered media using dyadic Green's functions and the scattering matrix method. *Journal of Quantitative Spectroscopy and Radiative Transfer* **110**, 2002–2018 (2009).

30. Fu, C. J. & Zhang, Z. M. Nanoscale radiation heat transfer for silicon at different doping levels. *International Journal of Heat and Mass Transfer* **49**, 1703–1718 (2006).

31. Spitzer, W. G. & Fan, H. Y. Determination of optical constants and carrier effective mass of semiconductors. *Physical Review* **106**, 882–890 (1957).

32. Macfarlane, G. G., McLean, T. P., Quarrington, J. E. & Roberts, V. Fine structure in the absorption-edge spectrum of Si. *Physical Review* **111**, 1245–1254 (1958).

33. Morin, F. J. & Maita, J. P. Electrical properties of silicon containing arsenic and boron. *Physical Review* **96**, 28–35 (1954).

34. Sze, S. M. *Physics of Semiconductor Devices* (John Wiley & Sons, New York, 1981).

35. Songprakob, W., Zallen, R., Liu, W. & Bacher, K. Infrared studies of hole-plasmon excitations in heavily-doped p-type MBE-grown GaAs: C. *Physical Review B - Condensed Matter and Materials Physics* **62**, 4501–4510 (2000).

36. Sze, S. M. & Ng, K. K. *Physics of Semiconductor Devices* (John Wiley & Sons, New York, 2006).

37. Littau, K. A. *et al.* Microbead-separated thermionic energy converter with enhanced emission current. *Physical Chemistry Chemical Physics* **15**, 14442–14446 (2013).

38. Lee, J. H., Bargatin, I., Melosh, N. A. & Howe, R. T. Optimal emitter-collector gap for thermionic energy converters. *Applied Physics Letters* **100**, 173904 (2012)
